# Whole-genome sequencing to delineate *Mycobacterium tuberculosis* outbreaks: a retrospective observational study

**DOI:** 10.1016/S1473-3099(12)70277-3

**Published:** 2013-02

**Authors:** Timothy M Walker, Camilla LC Ip, Ruth H Harrell, Jason T Evans, Georgia Kapatai, Martin J Dedicoat, David W Eyre, Daniel J Wilson, Peter M Hawkey, Derrick W Crook, Julian Parkhill, David Harris, A Sarah Walker, Rory Bowden, Philip Monk, E Grace Smith, Tim EA Peto

**Affiliations:** a Nuffield Department of Medicine, John Radcliffe Hospital, University of Oxford, Oxford, UK; b Department of Statistics, University of Oxford, Oxford, UK; c West Midlands Public Health Laboratory, Health Protection Agency, Heart of England NHS Foundation Trust, Birmingham, UK; d Heartlands Hospital and Birmingham Chest Clinic, Heart of England NHS Foundation Trust, Birmingham, UK; e Oxford National Institute of Health Research Biomedical Research Centre, John Radcliffe Hospital, Headington, Oxford, UK; f School of Immunity and Infection, University of Birmingham, Birmingham, UK; g Wellcome Trust Sanger Institute, Genome Campus, Hinxton, Cambridge, UK; h Health Protection Agency, County Hall, Glenfield, Leicester, UK

## Abstract

**Background:**

Tuberculosis incidence in the UK has risen in the past decade. Disease control depends on epidemiological data, which can be difficult to obtain. Whole-genome sequencing can detect microevolution within *Mycobacterium tuberculosis* strains. We aimed to estimate the genetic diversity of related *M tuberculosis* strains in the UK Midlands and to investigate how this measurement might be used to investigate community outbreaks.

**Methods:**

In a retrospective observational study, we used Illumina technology to sequence *M tuberculosis* genomes from an archive of frozen cultures. We characterised isolates into four groups: cross-sectional, longitudinal, household, and community. We measured pairwise nucleotide differences within hosts and between hosts in household outbreaks and estimated the rate of change in DNA sequences. We used the findings to interpret network diagrams constructed from 11 community clusters derived from mycobacterial interspersed repetitive-unit–variable-number tandem-repeat data.

**Findings:**

We sequenced 390 separate isolates from 254 patients, including representatives from all five major lineages of *M tuberculosis*. The estimated rate of change in DNA sequences was 0·5 single nucleotide polymorphisms (SNPs) per genome per year (95% CI 0·3–0·7) in longitudinal isolates from 30 individuals and 25 families. Divergence is rarely higher than five SNPs in 3 years. 109 (96%) of 114 paired isolates from individuals and households differed by five or fewer SNPs. More than five SNPs separated isolates from none of 69 epidemiologically linked patients, two (15%) of 13 possibly linked patients, and 13 (17%) of 75 epidemiologically unlinked patients (three-way comparison exact p<0·0001). Genetic trees and clinical and epidemiological data suggest that super-spreaders were present in two community clusters.

**Interpretation:**

Whole-genome sequencing can delineate outbreaks of tuberculosis and allows inference about direction of transmission between cases. The technique could identify super-spreaders and predict the existence of undiagnosed cases, potentially leading to early treatment of infectious patients and their contacts.

**Funding:**

Medical Research Council, Wellcome Trust, National Institute for Health Research, and the Health Protection Agency.

## Introduction

Control of *Mycobacterium tuberculosis* can be challenging even in high-income countries. Between 2001 and 2011, incidence of tuberculosis in the UK rose from 11·6 to 14·4 cases per 100 000 people per year,^1^ with active disease developing in individuals born outside the UK accounting for the increase.^2^ Detection of tuberculosis outbreaks is guided by mycobacterial interspersed repetitive-unit–variable-number tandem-repeat (MIRU-VNTR) genotyping.^1^ Although transmission between individuals infected with different genotypes can be excluded with this approach, epidemiological data are needed to confirm outbreaks when genotypes match.^3^, ^4^ Collection of such data is difficult if patients are unwilling or unable to volunteer information, as is commonly the case in some of the social groups most at risk of tuberculosis.^5^, ^6^ Even when genotyping does lead to outbreak detection, it offers no insights into the underlying pattern of transmission.

Whole-genome sequencing is an increasingly accessible and affordable alternative to MIRU-VNTR genotyping that can detect microevolution within *M tuberculosis* lineages as they are transmitted between hosts.^7^, ^8^, ^9^, ^10^ Because backwards mutations are rare,^11^ the pattern of accumulated mutations can theoretically suggest direction of transmission during an outbreak. Although whole-genome sequencing has a greater resolution than does MIRU-VNTR genotyping (as established in one specific outbreak),^12^ its full public health potential remains to be investigated.

In this study, our main aim was to estimate the genetic diversity of related strains of *M tuberculosis* in the Midlands region of the UK and to investigate where and how our measure of genetic diversity might be used to assess community outbreaks in detail. The region includes the cities of Birmingham and Leicester, where all five clades (lineages) of *M tuberculosis* are found in its ethnically diverse population.^13^, ^14^ Annual incidence of tuberculosis in these cities is up to 50–70 cases per 100 000 individuals.^1^

## Methods

### Study design

We sequenced isolates of *M tuberculosis* from an archive of more than 13 000 frozen cultures obtained between 1994 and 2011 that is held at the UK Health Protection Agency (HPA) Regional Mycobacterial Reference Laboratory for the Midlands, South Yorkshire, and Humberside (Heartlands Hospital NHS Foundation Trust, Birmingham, UK).

We selected isolates to estimate genomic diversity within and between hosts and categorised them into four groups. First, we estimated within-host cross-sectional diversity from paired pulmonary and extrapulmonary isolates received within 1 month of each other. Cross-sectional samples were selected at random until 50 pairs had been successfully located, cultured, and prepared for whole-genome sequencing. Second, we measured within-host longitudinal diversity, with two or more pulmonary isolates from the same patient separated by at least 6 months. We intended to select 100 longitudinal isolates, preferentially those from patients with the largest intervals between samples. Third, we estimated between-host diversity in a transmission chain from household outbreaks. We selected all isolates from household outbreaks identified by the reference laboratory. Fourth, we measured diversity within community-based MIRU-VNTR-defined clusters (including school clusters) to specifically investigate what additional benefits whole-genome sequences might provide as compared with MIRU-VTNR. We selected eleven MIRU-VNTR-based community clusters (six to 47 patients) identified by public health teams as containing some cases in which direct case-to-case transmission was supported and others where it was uncertain. These clusters were matched at 15 or 24 MIRU-VTNR loci according to the typing protocol at the time of referral. To relate school clusters to their community, we added local 24-locus MIRU-VNTR matching cases when available. To investigate potential clustering across 24-locus MIRU-VNTR types, some clusters were extended to include isolates that did not match at up to two loci.

In England and Wales, some diseases have to be reported to local authorities (Public Health Act 1984). Public health action taken as a result of notification and surveillance is one of the HPA's key roles and the 2003 HPA Act and the 2002 Statutory Instrument 1438 provide the legislative cover to undertake necessary follow-up. Part of this follow-up is identification of links between cases, which is made possible by increasingly robust methods. A UK Clinical Research Collaboration grant enabled whole-genome sequencing, which offered the potential to detect genetic differences with improved resolution to further identify possible case links or refute such links on the basis of strain divergence. Such methods will increasingly be used in the future and the purpose of the grant was to see how useful such methods would be in practice now. Less robust methods to identify genetic differences were already in routine use in laboratories and it was therefore argued that, as a service-delivery assessment, a research ethics committee application was not warranted for this work.

### Procedures

We took cultures from Löwenstein-Jensen slopes as confluent growth and purified and extracted DNA as described previously.^15^ Samples were sequenced on the Illumina HiSeq platform (Illumina, San Diego, CA, USA) at the Wellcome Trust Sanger Institute (Hinxton, UK) to produce 75 base-pair paired-end reads that were mapped with Stampy (version 1.0.13),^16^ without Burrows-Wheeler Aligner premapping, with an expected substitution rate of 0·01 to the H37Rv (GenBank NC000962.2) reference genome. A consensus of more than 75% of reads was necessary to support high-confidence nucleotide variant calls made with SAMtools mpileup,^17^ which had to be homozygous in a diploid model. Only variants supported by at least five reads, including one in each direction that did not occur at sites with unusual depth and were not within 12 base pairs of another nucleotide variant or indel, were accepted.^18^, ^19^ Consistency of the sequencing, assembly, and data filtering process was assessed by resequencing isolates from 23 patients and the H37Rv reference genome on different flow cells to produce 59 technical replicates.

We estimated the molecular clock (ie, the rate of change in the whole genome sequence) by use of maximum likelihood from longitudinal pairs of isolates within individuals and from households with a coalescent model,^20^ assuming a Poisson distribution for the accumulation of mutations. We obtained CIs by parametric bootstrap. Maximum-likelihood trees were constructed from concatenated variable sites across clustered genomes with PhyML 3.0 in Seaview,^21^ and were rooted with other isolates in the collection. Uncalled sites where variants had been identified in other samples were manually inspected and nucleotides initially excluded because of excessive (>97·5th percentile) high-quality read depth were reinserted in an additional filtering step.^22^

Clinical, demographic, and microbiological data were available for all isolates. We obtained epidemiological data in interviews with public health teams and supplemented the data by case-record review. UK guidelines for contact tracing recommend screening household contacts for every new index case, at-risk individuals, and any other named contacts in the community when the index case is thought to be infectious.^23^ Epidemiological relations within clusters were graded as linked (ie, patients had shared time and space with each other or a third party), possibly linked (ie, known to have shared space, but not at the same time), or no known link (ie, no known shared space). We summarised epidemiological relations, differences in single nucleotide polymorphisms (SNPs), and differences in isolation date in network diagrams representing patients (through their sequences) as nodes. In each cluster, starting with the first patient to be diagnosed, epidemiologically linked patients (or nodes) were joined by edges. The most parsimonious number of connecting edges (ie, number of nodes minus one) needed to represent the most plausible transmission network within each cluster was chosen. To minimise the SNP distance between any one patient's isolates and those of other patients in the cluster, we used one isolate per patient when subsequent isolates were the same or a greater distance away. We used two isolates from one patient when some patients were closer to the first and others to the second. When more than one epidemiologically linked case was identified, and after all epidemiologically linked cases in the network had been connected, edges were chosen to minimise SNP difference and then time interval between isolates.

Although school outbreaks are often caused by particularly infectious individuals leading to many secondary cases,^24^ one important question is whether so-called super-spreaders are also the source of many community outbreaks.^12^ Because *M tuberculosis* evolves by descent,^25^, ^26^ there is an a-priori expectation that a star-like phylogenetic topology with several secondary cases branching directly from a common node would be apparent when an individual with several contacts remains infectious for some time. We used this approach to investigate whether super-spreaders were evident in our community clusters.

### Role of the funding source

The sponsors of the study had no role in study design, data collection, data analysis, data interpretation, or writing of the report. The corresponding author had full access to all the data in the study and had final responsibility for the decision to submit for publication.

## Results

**Figure 1:**
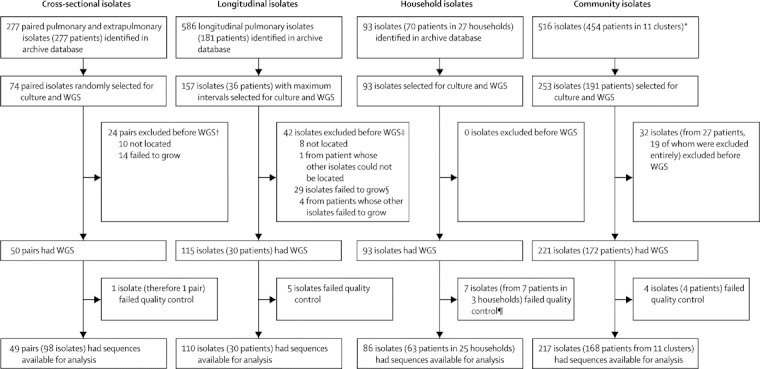
Sample selection The cross-sectional and community analyses datasets overlapped by 14 isolates (eight patients); the longitudinal and community analyses by 23 (five); the longitudinal, household, and community analyses by 26 (seven); and the household and community analyses by 32 (29). WGS=whole-genome sequencing. *Cluster 9 is a large, previously described cluster defined by mycobacterial interspersed repetitive-unit–variable-number tandem-repeat genotyping that we did not attempt to sequence completely because of its size (>280 patients);^27^ 18 patients from this cluster were included because 46 isolates from them had been sequenced as cross-sectional, longitudinal, or household isolates; in the remaining ten community clusters, we attempted to culture and sequence all 207 isolates (173 patients), successfully sequencing 171 isolates (150 patients). †Mean time since original isolation was 8 years for missing isolates and 9 years for isolates that failed to regrow, compared with 5 years for successfully cultured isolates. ‡Mean time since original isolation was 10 years for missing isolates and 8 years for isolates that failed to regrow, compared with 6 years for successfully cultured isolates. §One patient excluded because all his or her isolates failed to grow. ¶Only two households were excluded.

We successfully sequenced 390 separate isolates from 254 patients (figure 1), including representatives from all global lineages (European-American and central Asian lineages occurred most frequently; appendix) and those infected with *Mycobacterium bovis* and *Mycobacterium africanum*.^14^ Mean reference genome coverage was 88·5% (range 86·5–89·5%). No discrepancies between base-calls for the 59 technical replicates were recorded.

The mean number of isolates from each of the 30 patients for whom we had sequences of longitudinal isolates available for analysis was 3·7 (range 2–9). The mean length of time between the first and last isolate was 30 months (6–102). For the household isolates, 25 households each provided a mean of 3·4 isolates (2–19) to the analyses from a mean of 2·5 patients (2–5). The household outbreaks lasted a mean of 20 months (0–125). Of community clusters, three were characterised by school outbreaks, six by community-based substance misuse, one by a regionally dispersed ethnic group, and one by *M bovis* infection (table). Mean duration of community outbreaks was 81 months (range 9–144).

**Table:** MIRU-VNTR-based community clusters

	**Dominant MIRU-VNTR profile**	Phylotype*	**Description**	**Patients linked to cluster**			**WGS isolates**	**Time span of WGS isolates (months)**	**Known links between patients**†			**Possible links between patients**†			**No known links between patients**†		
				By MIRU-VNTR 15	By MIRU-VNTR 24	By discordant MIRU-VNTR (22-23 loci)			≤5 SNPs	6–12 SNPs	>12 SNPs	≤5 SNPs	6–12 SNPs	>12 SNPs	≤5 SNPs	6–12 SNPs	>12 SNPs
Cluster 1	32433.2332514327.223423352	European-American	School	0	8	0	9	13	7	0	0	0	0	0	0	0	0
Cluster 2	32333.2512515324.234433363	European-American	School	6	3	0	9	92	6	0	0	1	0	0	1	0	0
Cluster 3	42234.2742511324.432423254	Central Asian	School	0	6	0	6	59	4	0	0	0	0	0	0	0	1
Cluster 4	32433.232515322.224423542	European-American	Substance misuse	15	31	1	54	88	8	0	0	0	0	0	38	0	0
Cluster 5	32433.2432515323.241433273	European-American	Substance misuse	7	2	0	9	138	1	0	0	6	0	0	1	0	0
Cluster 6	32433.2432515324.443443153	European-American	Substance misuse	3	19	4	29	144	20	0	0	1	1	0	1	2	0
Cluster 7	−2234.2742511334.432422254	Central Asian	Substance misuse	0	8	2	17	104	7	0	0	1	1	0	0	0	0
Cluster 8	42435.2332517333.346443584	Beijing	Substance misuse	0	6	0	6	9	1	0	0	1	0	0	3	0	0
Cluster 9	32333.2432515314.434443183	Unknown‡	Substance misuse	0	16	2	46	83	7	0	0	0	0	0	7	3	0
Cluster 10	42234.2742511334.432423254	Central Asian	Ethnic group	2	21	0	26	102	6	0	0	0	0	0	10	0	6
Cluster 11	75553.2222415322.234323241	*Mycobacterium bovis*	..	1	5	0	6	60	2	0	0	1	0	0	1	1	0
Total	..	..	..	34	125	9	217	..	69	0	0	11	2	0	62	6	7

Data are numbers unless otherwise stated. MIRU-VNTR=mycobacterial interspersed repetitive-unit–variable-number tandem-repeat. WGS=whole-genome sequencing.

* Phylotypes based on the schema approved by the Health Protection Agency.^14^

† Total number of links in a cluster=total number of patients in cluster sequenced minus one.

‡ Spoligotyped as European-American.^27^

**Figure 2:**
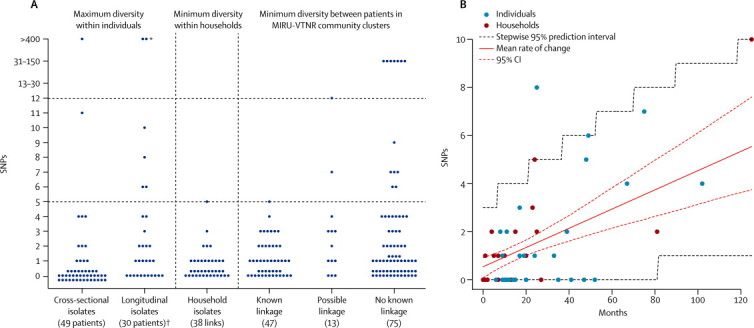
Genetic diversity of related isolates of *Mycobacterium tuberculosis* (A) Time-unadjusted pairwise genetic distances in SNPs. 22 of the 38 links within the 25 household clusters also occur within community clusters (ie, known linkage) but are shown with household isolates and not with community isolates. Top horizontal dashed line indicates the threshold above which direct transmission can be judged to be unlikely; bottom horizontal dashed line indicates the threshold below which transmission should be investigated. (B) Rate of change in DNA sequences estimated by coalescent-based maximum likelihood from the first and last isolates from individuals with persistent open tuberculosis and from households. SNP=single nucleotide polymorphism. MIRU-VNTR=mycobacterial interspersed repetitive-unit–variable-number tandem-repeat.*Isolates had substantially different MIRU-VNTR profiles. †Pair of *Mycobacterium africanum* isolates are represented two SNPs apart.

The greatest genomic diversity expected within individuals was estimated by sequencing paired (pulmonary *vs* non-pulmonary) isolates from 49 patients and 110 longitudinal isolates from 30 patients (figure 2). In three cases, the large numbers of SNPs that separated isolates (475, 1032, and 1096; figure 2) suggested that patients had been secondarily infected with a different strain rather than within-host evolution. In one patient with tuberculous meningitis and normal chest CT, paired cerebrospinal fluid and sputum isolates differed by 11 SNPs (figure 2). In four individuals who developed drug resistance during 7–10 years of persistent pulmonary infection, maximum genetic distance ranged from six to ten SNPs (figure 2). All 71 other pairwise comparisons (ie, cross-section and longitudinal isolates) differed by up to five SNPs (figure 2). Cross-sectional isolates had significantly fewer SNPs than did longitudinal isolates (37 [79%] of 47 *vs* 11 [46%] of 24 had no SNPs; rank-sum p=0·009; figure 2).

We estimated the genetic distances between individuals in known recent transmission chains from the sequences of 86 isolates from 63 individuals in 25 household-defined outbreaks. All 38 links (number of patients minus number of outbreaks) between patients were five or fewer SNPs. We recorded no evidence that the distribution of SNPs below this threshold differed from that of longitudinal isolates (38 [100%] of 38 *vs* 24 [80%] of 30; rank-sum p=0·16; figure 2). Overall, with exclusion of differences of more than 400 SNPs, 109 (96%) of 114 paired isolates from within individuals and household outbreaks differed by five or fewer SNPs, 108 (95%) by four or fewer SNPs, and 103 (90%) by three or fewer SNPs.

We estimated the rate of microevolution of *M tuberculosis* from the first and last sequenced isolates of the longitudinally sampled patients and from the household outbreaks. A rate of change in DNA sequences of 0·5 SNPs per genome per year (95% CI 0·3–0·7) was inferred by maximum likelihood (figure 2). However, within the variation we recorded, some rates were consistent with latent infection. All longitudinally sampled patients received antituberculosis drug treatment. HIV testing was not systematically done until 2011 (only eight results were available, all negative); however, UK rates of co-infection are fairly low, declining steadily from 9·0% in 2003, to 4·9% in 2010.^28^ We recorded weak evidence that the initial genetic diversity and rate of change in DNA sequences might vary between household outbreaks and longitudinally sampled individuals (joint *vs* separate models, p=0·08). However, the mutation rate was lower in individuals followed up longitudinally (0·3 SNPs per genome per year [95% CI 0–0·6]) than in those in household outbreaks (0·6 SNPs per genome per year [0·3–0·9]), and the initial diversity was higher (1·2 SNPs [0·3–1·9] *vs* 0·2 SNPs [0·008–0·7]).

We used these results to construct two thresholds against which to assess the MIRU-VNTR-based community clusters. We expected epidemiological linkage consistent with transmission to exist between isolates differing by five or fewer SNPs, and not to exist between isolates differing by more than 12 SNPs. We deemed pairs differing by six to 12 SNPs to be indeterminate.

The 11 community clusters were defined by their MIRU-VNTR profile (or up to two locus mismatches; table). Starting from the first case in each cluster, we constructed 11 networks (one for each cluster), accounting for 157 potential transmissions (edges; appendix). Within the three clusters centred on schools, 17 (85%) of 20 patients could be epidemiologically linked (table), with no link confirmed in three MIRU-VNTR-matched community isolates (the community-based case in cluster three was 35 SNPs away from the school isolates). In clusters six and seven, 27 (79%) of 34 patients could also be epidemiologically linked (table). However, in the remaining six clusters, including one associated with an African immigrant community (cluster ten), only 25 (24%) of 103 patients could be epidemiologically linked (table).

None of 69 epidemiologically linked and two (15%) of 13 possibly epidemiology linked patients were separated by more than five SNPs (seven and 12 SNPs respectively); conversely, 13 (17%) of 75 epidemiologically unlinked patients were separated by more than five SNPs and seven (9%) by more than 12 SNPs (three-way comparison exact p<0·0001; table). However, 22 potential transmissions of the 69 known to be epidemiologically linked featured in both household and community outbreaks. Excluding those, the number of epidemiologically linked patients differing by five SNPs or fewer was 47 (p=0·003).

The ability to identify cryptic outbreaks was most evident in cluster four, in which five SNPs or fewer separated 38 individuals with a background of substance misuse for whom contact tracing had been difficult (table). The ability to rule out transmission was particularly evident in cluster ten, in which more than 30 SNPs separated five individuals from a recent immigrant community and one British-born individual from the next nearest patient. In this cluster, isolates from ten patients from two cities 45 miles apart, with no known epidemiological links, could be genetically linked by five or fewer SNPs.

**Figure 3:**
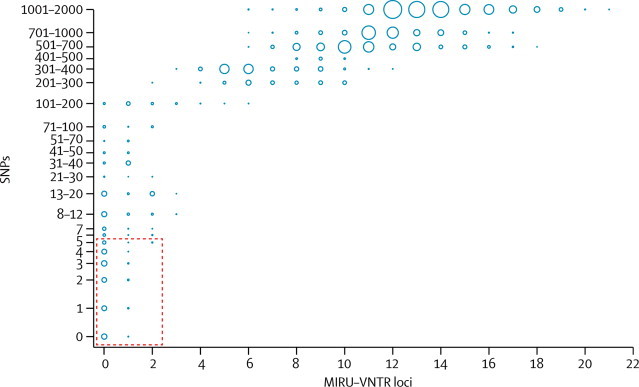
SNPs between MIRU-VNTR types by number of locus differences Comparison of all isolates with complete 24-locus MIRU-VNTR profiles. As each isolate was compared to each other isolate, the number of SNPs and MIRU-VNTR loci at which they diverge was recorded. Results are plotted on a log scale. Circle sizes are proportionate to the number of pairs diverging by a specific number of loci and SNPs. Dashed red box includes isolates that differ by five or fewer SNPs. SNP=single nucleotide polymorphism. MIRU-VNTR=mycobacterial interspersed repetitive-unit–variable-number tandem-repeat.

To explore the potential for 24-locus MIRU-VNTR typing to miss genuine transmission, we assessed the proportion of sequenced isolates matching only at 22 or 23 MIRU-VNTR that were genetically linked by five or fewer SNPs. Of 195 isolates typed at 24 loci, 14 pairs were matched at 22–23 loci. Three pairs of isolates were from individuals and three from households, all within one SNP of each other. Of four mismatching isolates in community clusters, two had possible epidemiological links to the cluster (four and 12 SNPs) and two had no known link to the cluster (none and five SNPs). The remaining four pairs had no known epidemiological links and were separated by 25, 94, 96, and 275 SNPs (exact p=0·03 comparing six of six known links [individuals and households] *vs* three of eight other links of five or fewer SNPs). No isolates matching at up to 21 MIRU-VNTR loci were genetically linked by five or fewer SNPs (figure 3, appendix).

**Figure 4:**
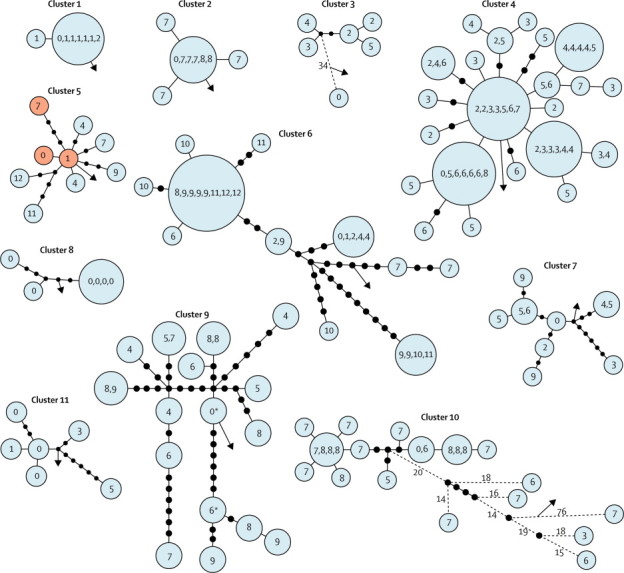
Genetic distances within 11 community clusters Genetic distances estimated with maximum likelihood. Each blue circle represents a node of people who were infected with isolates separated by no SNPs. Each number within a circle is one patient, the number indicates at which year during the outbreak they were diagnosed (the first infected is represented by 0). For patients with several isolates, the closest in SNPs to the next patient is included. Black circles are added when patients within blue circles are separated by more than one SNP; one black circle represents a difference of one SNP. Dashed lines in clusters three and ten show larger SNP distances (not to scale), with numbers representing the SNP difference. Arrows indicate the next closest isolate in the sequenced collection. Cluster five has three red nodes that were sequenced after the blue nodes; the existence of the central red node was suggested by the constellation of surrounding blue nodes. SNP=single nucleotide polymorphism. *Two isolates from one patient.

**Figure 5:**
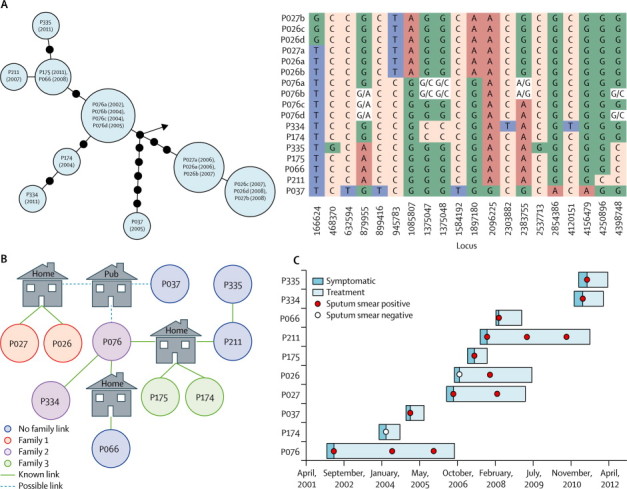
Detailed investigation of cluster seven Genetic tree and matrix of nucleotide variants (A). Genetic distances estimated with maximum likelihood. Each blue circle represents a node of people who were infected with isolates separated by no single nucleotide polymorphisms. Numbers within nodes are patient numbers and years of sample isolation are given in parentheses. The matrix shows nucleotide variants. Epidemiological network (B). Time of onset of symptoms, diagnosis, and treatment (C). Sputum smear positive samples show probable infectious periods.

The star-like pattern that would be expected with a super-spreader was apparent in all non-school outbreaks except for cluster eight (figure 4). The possible presence of a super-spreader was supported clinically and epidemiologically in clusters five and seven. In cluster five, the nine isolates were sequenced in two separate HiSeq runs. Phylogenetic analysis of the first six isolates identified a vacant central node in the genetic tree, which is consistent with a potentially still unsequenced common root (isolate). One of the three isolates from the subsequent HiSeq run matched this predicted sequence precisely (figure 4). The isolate belonged to a treatment non-compliant drug dealer with cavitating, pulmonary, smear-positive tuberculosis. This probable super-spreader had been diagnosed early in the outbreak, had interacted with many contacts, and was eventually detained under public health law in the interests of public safety. In cluster seven, the centrally placed individual was treatment non-compliant for 4 years, and had cavitating, pulmonary, smear-positive disease and known or possible epidemiological links to all other infected individuals. Notably, variants in other patients' sequences were present as mixed base-calls in this individual's four sequenced genomes (figure 5). Insufficient epidemiological data for the presence of a super-spreader were available for other clusters.

In addition to identifying super-spreaders, phylogenetic topology was indicative of the sampling density within MIRU-VNTR clusters. For example, fewer vacant black nodes can be seen in cluster four, where isolates from 47 (90%) of 52 patients clustered by MIRU-VNTR were sequenced, than in cluster nine, where only 18 (6%) of 280 were sequenced (figure 4).

## Discussion

Whole-genome sequencing can delineate the margins and structure of tuberculosis outbreaks with unprecedented resolution (panel). We established that most *M tuberculosis* isolates were within five SNPs of another isolate taken from the same individual or from a household contact. This finding provides essential context for interpretation of the diversity expected from closely related isolates in transmission chains. In a setting with low HIV prevalence, we estimated that the rate of genetic changes was 0·5 SNPs per genome per year, which is similar to estimates in macaques.^29^ We predicted that the maximum number of genetic changes at 3 years would be five SNPs and at 10 years would be ten SNPs. We then showed that all epidemiologically linked patients could be genetically linked by five or fewer SNPs. Our results suggest that within MIRU-VNTR-defined clusters, whole-genome sequencing offers sufficient resolution to identify or discount outbreaks.

Panel **Research in context**

**Systematic review**

We searched PubMed with the key words “tuberculosis”, “whole genome sequencing”, “outbreak”, and “cluster” for reports published in English before June 15, 2012. When our study began, only one report of the application of whole-genome sequencing to analysis of *Mycobacterium tuberculosis* outbreaks had been published.^8^ Schürch and colleagues^8^ presented data from a small subset of isolates belonging to a larger cluster and showed the step-wise accumulation of single nucleotide polymorphisms between transmitting individuals with time. A second, larger study was reported in 2011,^12^ and detailed the use of whole-genome sequencing in combination with a social network analysis for the investigation of an entire cluster. Gardy and colleagues^12^ showed that resolution of whole-genome sequencing was greater than was that of mycobacterial interspersed repetitive-unit–variable-number tandem-repeat (MIRU-VNTR) typing by delineating two outbreaks with a MIRU-VNTR-defined cluster. A third key report was published later in 2011,^29^ describing the molecular clock in macaques and giving the first indication of a mutation rate for *M tuberculosis*. Ford and coworkers^29^ presented data that suggested that the rate of change in DNA sequences was constant at 0·5 single nucleotide polymorphisms per genome per year in latent, active and re-activated disease.

**Interpretation**

Our study expands on previous findings to provide a framework for the analysis of whole-genome sequencing data as applied to field epidemiology. By assessing the genetic diversity within and between individuals, we have provided essential information for investigators attempting to apply this technology to real outbreaks. A key component of our analysis was an estimation of the molecular clock in people. We have shown how whole-genome sequencing technology can be used to distinguish between patients who are probably part of a recent transmission chain and those who are not. This ability to link patients to an outbreak before epidemiological data have been gathered could have substantial implications for contact tracing in the future. The apparent potential for whole-genome sequencing data to identify super-spreaders could affect these practices still further.

The greatest public health benefit is expected from tracing the contacts of the most infectious individuals. Within eight non-school community clusters, we identified two possible super-spreaders by inspecting the genetic trees for nodes from which several lineages diverged and by comparing this phylogenetic signal with epidemiological data. Although often hypothesised, direct genetic evidence for such super-spreaders has previously been elusive. In the two clusters with potential super-spreaders, the individual or isolate located in the centre of the tree was also epidemiologically the most probable source of secondary cases. The situation in cluster five is of particular interest. Patients who remain undiagnosed cannot be detected by current typing methods, have many opportunities to cause secondary infections, and, as the natural history of disease suggests, might never seek medical attention.^30^ Although in our specific examples the central node or source case was diagnosed early in each outbreak, public health teams could use data from whole-genome sequencing in real time to target active cases when the existence of undiagnosed individuals is predicted by the evolving genetic topology of the outbreak. As interpretation of the topology of genetic trees becomes increasingly robust, it might become not only a useful instrument for identification of potential super-spreaders, but also for assessment of the completeness of outbreak investigations and likelihood of continuing transmission.

Although MIRU-VNTR typing has been an effective way to identify outbreaks of tuberculosis,^3^, ^4^, ^27^, ^31^ the genomic diversity within some MIRU-VNTR genotypes^11^, ^32^ can leave public health teams uncertain about how intensively to investigate clusters when epidemiological links are not apparent. Our results, which support the evidence that microevolutionary events can change a MIRU-VNTR genotype within a host,^33^ add to this uncertainty. The effects are that long and costly contact tracing efforts could ultimately be futile. By contrast, the reliability of whole-genome sequencing can provide impetus for targeted contact tracing and interventions. For example, these data informed investigations into cluster seven, helping to identify additional contacts who then received isoniazid and rifampicin prophylaxis after testing positive for latent disease by interferon-γ release assays (unpublished).

As well as the molecular clock, our thresholds of five or fewer or more than 12 SNPs are relevant beyond the setting from which they were derived. Because our study population was ethnically diverse and produced isolates of all five *M tuberculosis* clades, and many people originated from high-transmission countries, we believe these results will be valid in high-incidence settings outside of the Midlands, UK. However, the usefulness of these findings in these settings still needs to be formally assessed. Patients who remain undiagnosed or receive inadequate treatment could transmit tuberculosis as super-spreaders, leading to increased cluster sizes. Public health teams could derive more benefit from ruling out recent transmission (>12 SNPs) than they do from relating cases to each other by five or fewer SNPs, because the number of intermediary cases within 3 years of evolution might still be large. Nevertheless, identification of phylogenetic signals for potential super-spreaders should be possible in such settings. As indicated by the mixed base-calls recorded in cluster seven, several mycobacterial subpopulations could arise within inadequately treated patients, potentially aiding the identification of super-spreaders. Although characterisation of the dynamics of cluster nine (>280 patients) would therefore have been informative, we did not have the resources to sequence it in its entirety. However, the phylogenetic topology in cluster four is consistent with more than one super-spreader, although the epidemiological history of this outbreak was insufficiently characterised to allow us to draw any further conclusions.

A potential limitation of our study is that we did not sequence all isolates from the community-based clusters because some were unavailable. However, when we sequenced isolates that diverged by five or fewer SNPs, any missing intermediate cases would make transmission more not less plausible. Additionally, real-time contact investigations have to deal with missing data, using existing information to judge the plausibility of additional, intermediary cases. Another limitation is that we could accurately map only about 88% of each genome, excluding the repetitive segments within the reference genome that include the regions defining MIRU-VNTR. Although additional diversity could therefore be masked from our analysis, how much additional resolution this diversity would provide is unclear.

In view of the rapidly declining costs of whole-genome sequencing and advances that have substantially improved turnaround-times,^7^ the HPA is considering its introduction for routine tuberculosis public health practice in England (unpublished). Costs are approaching GBP£50 per sequence, which are similar to those of MIRU-VNTR typing. Whole-genome sequencing will probably be of greatest benefit in complex community outbreaks when epidemiological data are difficult to obtain and the potential for targeted and effective public health surveillance and intervention is substantial. Our findings are therefore an early indication of the potential for whole-genome sequencing to transform tuberculosis prevention and control.
