# Supplementary material for: Whole-genome sequencing to delineate Mycobacterium tuberculosis outbreaks: a retrospective observational study
Source: Lancet Infect Dis. 2013 Feb;13(2):137–46. doi: 10.1016/S1473-3099(12)70277-3 (PMC3556524; doi:10.1016/S1473-3099(12)70277-3)

THE LANCET Infectious Diseases

Supplementary webappendix

This webappendix formed part of the original submission and has been peer reviewed. We post it as supplied by the authors.

Supplement to: Walker TM, Ip CLC, Harrell RH, et al. Whole-genome sequencing to delineate *Mycobacterium tuberculosis* outbreaks: a retrospective observational study. *Lancet Infect Dis* 2012; published online Nov 15. http://dx.doi.org/10.1016/S1473-3099(12)70277-3.

Supplementary appendix

Further details on sample selection:

We pre-specified four groups of isolates within which to investigate within and between host diversity: (i) cross-sectional diversity within individuals (pulmonary vs. non-pulmonary isolates within 6 months of each other), (ii) longitudinal diversity within individuals (patients with multiple pulmonary isolates >6 months apart), (iii) diversity between individuals in known household clusters, and (iv) MIRU-VNTR-based community clusters. As we had funding to sequence 400 to 500 samples, we attempted to sequence specific numbers of isolates within each group.

We first searched the archive database for all isolates within the above groups. From these groups, specific isolates were selected for sequencing as below. Where initially selected samples could not be found or did not grow, replacements were selected in the same way as the initial samples. In some cases the freezer location was not listed in the database so the isolate could not be retrieved; in other cases there was no vial present in the specified freezer location; other vials contained non-viable bacteria - unsurprising given the age of some of the samples.

(i) Cross-sectional paired isolates were chosen at random, until 50 pairs of DNA preparations ready for sequencing had been obtained (in total 74 pairs selected for locating in freezers and growing). As no data were available on likely within-host diversity at the time the choices were made, the target sample size was 50 to ensure that if no single nucleotide polymorphisms (SNPs) were observed between any pair, then the upper 97.5% confidence limit around the observation of 0% with ≥1 SNP was 7%.

(ii) Longitudinal samples within individuals were selected to maximize the time period between first and last isolate for each patient, as longitudinal diversity over time was considered to be most relevant to onward transmission. Any intervening samples were also included for sequencing from these patients. No data were available on likely diversity over time when the samples were chosen, so the arbitrary decision was made to sequence 100 isolates within this group, a similar number to those previously used to estimate molecular clock rates (Didelot X, Eyre D, Cule M *et. al.* Microevolutionary analysis of *Clostridium difficile* genomes to investigate transmission, *Genome Biology* 2012 (in press).

(iii) and (iv) We aimed to sequence all isolates from all household outbreaks known to the surveillance laboratory (total 93 isolates) and from 10 reasonably sized (6-47 patients) MIRU-VNTR-based community clusters identified by the public health teams as containing some cases where direct case-to-case transmission was supported and others where it was uncertain (total 207 isolates). (Note: in other groups (i), (ii), and (iii), 46 isolates were sequenced from 18 patients who also belonged to a very large MIRU-VNTR-defined cluster containing >280 patients (reference 27 in main paper); these 18 patients were analysed as an 11th cluster but selection for sequencing was not based on this cluster membership).

Of note, both failure to be located and failure to grow were strongly related to duration of storage. As expected, the longer the duration of storage the more likely an isolate could not be located, and, if it was located, that it failed to grow. For example, among the cross-sectional isolates the mean time since original isolation among the missing isolates was 8 years and among the isolates that failed to re-grow it was 9 years. This contrasted to 5 years for the successfully cultured isolates. Among the longitudinal isolates the mean time was 10 years for missing isolates and 8 years for those that failed to re-grow. It was 6 years for successfully cultured isolates. For groups (i), (ii) and (iii) missing data from one isolate sometimes meant that other sequences were also excluded (eg if one of two longitudinal isolates from a patient in (ii) could not be located/failed to grow, then the patient could not contribute any data to (ii)).

All missing data is assumed to be completely at random in the analysis, with sequenced cases assumed to represent the underlying population. This assumption was felt to be reasonable by the HPA referral laboratory as they have not seen any marked variation in type of cases over the last decade, so length of storage is therefore not a plausible confounder. The global lineages of sequenced strains reflect those prevalent in the Midlands, as

would be expected from essentially random sampling.

In real-world, real-time settings, detecting outbreaks is unlikely to suffer from the problem of missing, un-culturable or un-sequencable samples. No isolates will fail to grow as the sequenced cases will all be, by definition, culture positive. Growth failures in our study were strongly related to previously cultured isolates dying in the freezer or as a consequence of freeze-thawing. Sequencing failures will undoubtedly continue to occur but are much more likely when there is little DNA input (a consequence of poor growth) or where there is contamination (not a problem that is unique to sequencing).

S1. Maximum likelihood tree of 195 isolates typed at 24-MIRU-VNTR loci:

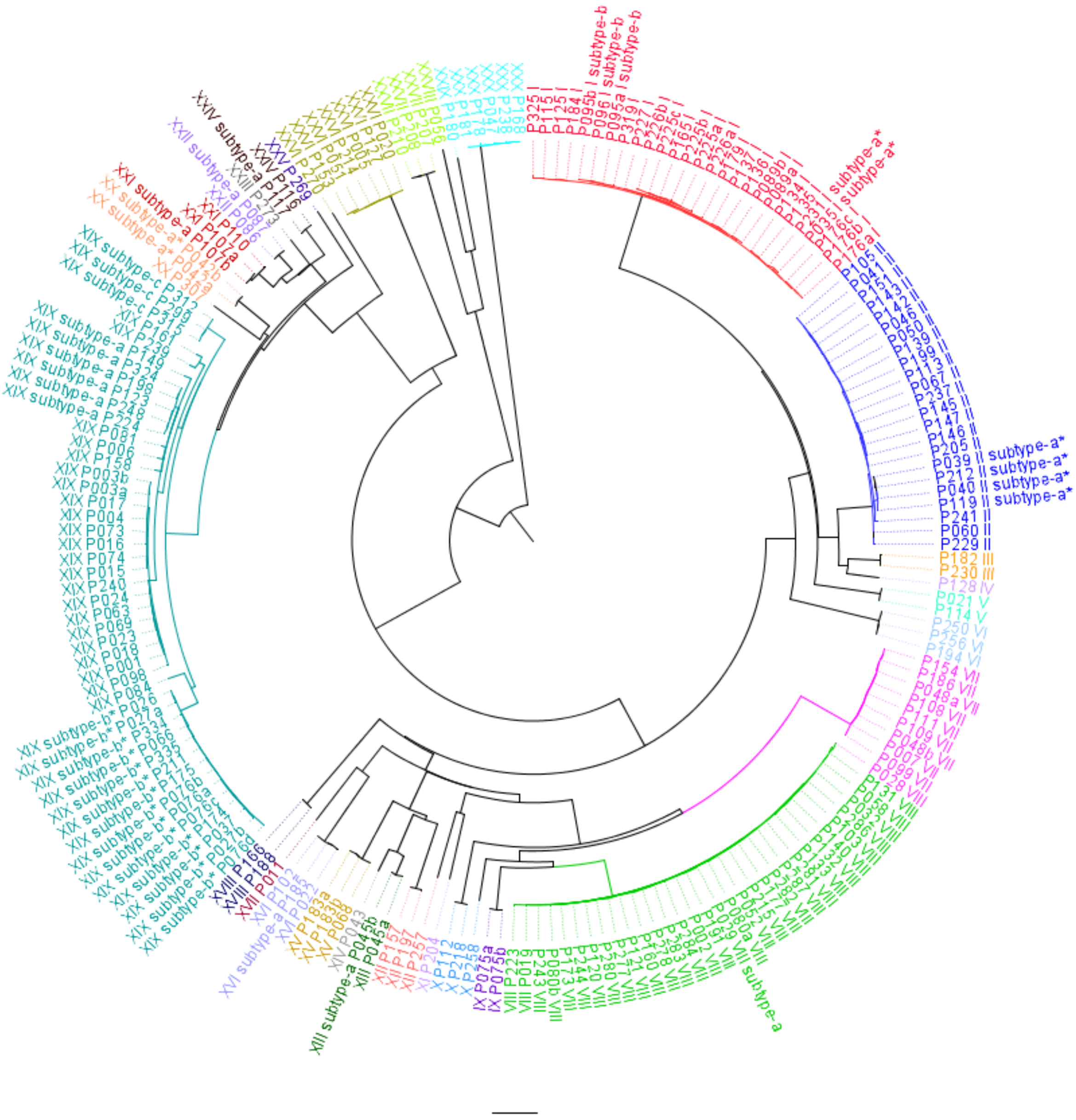

220 SNPs

MIRU-VNTR profiles and their subtypes are grouped by colour. Each isolate is labelled by a patient number (P) and by a roman numeral indicating the MIRU-VNTR type. Each subtype listed differs from the main type at a single MIRU-VNTR locus (* 2 loci where the subtype is asterisked).
Global MTB lineages by MIRU-VNTR type (based on HPA approved schema**):
Beijing: XXVI
European American: II-VIII, X-XVI
Central Asian: XVII, XIX-XXV
East-African Indian: XXVII-XXIX
Unassigned: I†, IX-X, XVIII, XXVI
M. Bovis XXX

** see reference 14 in main paper
† Isolates from this outbreak have been identified as part of the European American lineage by spoligotyping (see reference 27 in main paper)

S2. **Table of all isolates and patients appearing in each collection (C-1 = cross-sectional isolates from individuals, C-2 = longitudinal isolates from individuals, C-3 = longitudinal isolates from households, C-4 = MIRU-VNTR defined community clusters).**

Collection	Cluster / Household	Also appears in	European Nucleotide Archive (ENA) Accession number	Patient code	Year of isolation	VNTR	MIRU10	VNTR+	MIRU code for ML tree (S1)
C-1		C-4	ERR025837	P000002	2007	32433	2312515322	-24423542	VIII
C-1		C-4	ERR025838	P000002	2007				
C-1			ERR046889	P000008	2007	32234	2742511334		
C-1			ERR046936	P000008	2007				
C-1			ERR046848	P000009	2006	94464	2422622313		
C-1			ERR046898	P000009	2006	94465	2422622313		
C-1			ERR046897	P000011	2007	42234	2642517323	3124233b2	XVII
C-1			ERR046901	P000011	2007				
C-1			ERR025839	P000025	2008	42435	2232518333		
C-1			ERR025840	P000025	2008	42435	2232518333		
C-1			ERR046832	P000033	2007	61464	2432622134		
C-1			ERR046900	P000033	2007				
C-1		C-4	ERR046878	P000034	2006	32433	2312515322		
C-1		C-4	ERR046879	P000034	2006				
C-1			ERR038262	P000042	2008	42235	2642515333	442423384	XXa*
C-1			ERR038263	P000042	2008	42235	2642515333	442423384	XXa*
C-1			ERR046836	P000043	2007	21433	2412615221	234615242	XIV
C-1			ERR046837	P000043	2007	21433	2412615221		
C-1			ERR039330	P000045	2008	21433	2412615221	434415222	XIIIa
C-1			ERR046732	P000045	2008	21433	2412615221	434413222	XIII
C-1			ERR046846	P000049	2006	-2235	2632517333		
C-1			ERR046895	P000049	2006				
C-1			ERR038258	P000062	2008				
C-1			ERR038259	P000062	2008	54465	2432622313		
C-1			ERR046844	P000064	2008				
C-1			ERR046893	P000064	2008	84465	2431632313		
C-1		C-4	ERR046982	P000081	2007	4223-	2742511334	432423254	XIX
C-1			ERR046983	P000081	2007				
C-1			ERR046864	P000083	2006	42333	2532512323		
C-1			ERR046865	P000083	2006	42333	2532512323		
C-1			ERR046887	P000085	2006				
C-1			ERR046894	P000085	2006	22432	1432615327		
C-1			ERR046857	P000092	2006				
C-1			ERR046858	P000092	2006	42234	2-42511334		
C-1			ERR046882	P000097	2006				
C-1			ERR046883	P000097	2006	42235	2422517334		
C-1			ERR046884	P000100	2008				
C-1			ERR046885	P000100	2008	21433	2412815221		

C-1		C-4	ERR038254	P000101	2006	32433	2312515322		
C-1		C-4	ERR038255	P000101	2006				
C-1		C-4	ERR046876	P000124	2007	33433	2312515322		
C-1		C-4	ERR046877	P000124	2007	33433	2312515322		
C-1			ERR046833	P000138	2004	22433	2312514314		
C-1			ERR046888	P000138	2004				
C-1		C-4	ERR025833	P000149	2006	42234	2742511324	432423254	XIXa
C-1			ERR025834	P000149	2006				
C-1			ERR046862	P000152	2008	-2235	2522515333		
C-1			ERR046863	P000152	2008				
C-1			ERR046859	P000159	2006				
C-1			ERR046984	P000159	2006	32333	2432515324		
C-1			ERR046870	P000162	2008	21433	2412615221		
C-1			ERR046871	P000162	2008				
C-1			ERR046831	P000164	2007				
C-1			ERR046890	P000164	2007	22433	2322504322		
C-1		C-4	ERR046866	P000167	2007	42234	2742511334		
C-1		C-4	ERR046867	P000167	2007	42234	2742511334		
C-1			ERR046855	P000180	2007	64365	2432-22333	273313451	XXIX
C-1			ERR046856	P000180	2007				
C-1			ERR038260	P000201	2008				
C-1			ERR038261	P000201	2008	42235	2642513333		
C-1			ERR046849	P000202	2008	54436	2432-22333		
C-1			ERR046891	P000202	2008				
C-1			ERR046838	P000204	2007	22432	2332515323	232323372	XI
C-1			ERR046839	P000204	2007				
C-1			ERR046934	P000213	2006	41235	2522517333		
C-1			ERR046980	P000213	2006				
C-1			ERR046880	P000219	2006	84265	2432612413		
C-1			ERR046881	P000219	2006				
C-1			ERR025835	P000220	2007	32235	2542518333		
C-1			ERR025836	P000220	2007	32235	2542518333		
C-1			ERR046892	P000232	2007	-2453	2232515222		
C-1			ERR046935	P000232	2007	-2453	2232515222		
C-1			ERR046868	P000233	2007				
C-1			ERR046869	P000233	2007	32333	2432512322		
C-1			ERR046874	P000234	2003	32333	2532515323		
C-1			ERR046875	P000234	2003	32333	2532515323		
C-1			ERR046834	P000236	2007	42433	2431515321		
C-1			ERR046981	P000236	2007				
C-1			ERR046872	P000242	2003				
C-1			ERR046873	P000242	2003	32333	2531315323		
C-1			ERR046996	P000257	2008	42433	2331515323	442423384	XII
C-1			ERR047004	P000257	2008				
C-1			ERR046995	P000258	2007	32433	2312515324	223323432	X

C-1			ERR046999	P000258	2007				
C-1			ERR047000	P000260	2006	32333	2432515334		
C-1			ERR072028	P000260	2006				
C-1			ERR046997	P000261	2006				
C-1			ERR072039	P000261	2006	42433	2332315322		
C-1			ERR047003	P000262	2007				
C-1			ERR072027	P000262	2007	42435	2332517333		
C-1			ERR047001	P000268	2009	61467	2432622333		
C-1			ERR047002	P000268	2009	61467	2432622333		
C-1			ERR072021	P000269	2009	42235	2642515333	342423371	XXV
C-1			ERR072022	P000269	2009				
C-1			ERR072023	P000270	2007	22235	2562117333		
C-1			ERR072024	P000270	2007	22235	2562117333	442443395	XXVI
C-1		C-4	ERR046860	P000859	2008	42234	2742511334		
C-1		C-4	ERR046861	P000859	2008	42234	2742511334		
C-2		C-3 / 4	ERR040087	P000026	2006	-2234	2742511334	432422244	XIXb*
C-2		C-3 / 4	ERR046770	P000026	2008	-2234	2742511334		
C-2		C-3 / 4	ERR046940	P000026	2007				
C-2		C-3 / 4	ERR046942	P000026	2007				
C-2		C-3 / 4	ERR038298	P000027	2006	42234	2742511334	432422244	XIXb*
C-2		C-3 / 4	ERR046769	P000027	2008	-2234	2742511334	432422244	XIXb*
C-2			ERR025847	P000030	2000				
C-2			ERR025848	P000030	2002	32333	2531515323		
C-2			ERR039344	P000030	2003				
C-2		C-3 / 4	ERR046783	P000035	2008	32333	2432515315	434443182	Ia*
C-2		C-3 / 4	ERR072072	P000035	2010	42235	2642516333	442423382	XX
C-2		C-4	ERR046929	P000048	2009	32433	2332514327	223423352	VII
C-2		C-4	ERR046948	P000048	2008	32433	2332514327	223423352	VII
C-2			ERR046905	P000056	2005	61456	2432622334	374424562	XXVIII
C-2			ERR046907	P000056	2003				
C-2			ERR046915	P000056	2003	61456	2432722334		
C-2			ERR046918	P000059	2007	42432	2432424421		
C-2			ERR046933	P000059	2007	42432	2432424421		
C-2		C-4	ERR046825	P000071	2007				
C-2		C-4	ERR046937	P000071	2006				
C-2		C-4	ERR046943	P000071	2006				
C-2		C-4	ERR046986	P000071	2004				
C-2			ERR046776	P000075	2008	23433	2312505324	222423552	IX
C-2			ERR046777	P000075	2008				
C-2			ERR046778	P000075	2009	23433	2312505324	222423552	IX
C-2			ERR047005	P000075	2008				
C-2			ERR047006	P000075	2008				
C-2			ERR047008	P000075	2008				
C-2			ERR047016	P000075	2008				
C-2		C-4	ERR039323	P000076	2002	-2234	2742511334	432422254	XIXb

C-2		C-4	ERR046729	P000076	2004	-2234	2742511334	432422254	XIXb
C-2		C-4	ERR046772	P000076	2004	-2234	2742511334	432422254	XIXb
C-2		C-4	ERR046914	P000076	2005	-2234	2742511334	4324222--	XIXb
C-2		C-4	ERR038253	P000089	2006	32333	2432515314	434443183	I
C-2		C-4	ERR038256	P000089	2007	32333	2432515314	434443183	I
C-2		C-4	ERR038257	P000089	2008				
C-2		C-4	ERR039346	P000089	2005	32333	2422515314		
C-2		C-4	ERR046904	P000089	2008				
C-2		C-4	ERR046910	P000089	2006				
C-2		C-4	ERR046919	P000089	2006				
C-2			ERR039324	P000094	2004	42235	2642516333		
C-2			ERR046730	P000094	2006				
C-2			ERR046939	P000094	2002				
C-2			ERR046941	P000094	2003	42235	2642516333		
C-2		C-3 / 4	ERR046852	P000095	2004	32333	2432515314	43444318-	Ib
C-2		C-3 / 4	ERR046853	P000095	2006	32333	2432515314	433443183	Ib
C-2			ERR039325	P000128	2002				
C-2			ERR039326	P000128	2008				
C-2			ERR046906	P000128	2008				
C-2			ERR046912	P000128	2007	32523	2432515322	23444-493	IV
C-2			ERR046916	P000128	2005				
C-2			ERR046796	P000155	2005				
C-2			ERR046821	P000155	2001				
C-2			ERR046903	P000155	2004				
C-2			ERR025842	P000156	2000				
C-2			ERR025843	P000156	2001				
C-2			ERR025844	P000156	2004	32433	2512511323		
C-2			ERR025846	P000156	1998				
C-2			ERR039345	P000156	2003				
C-2			ERR046922	P000156	2006	32433	2512511323		
C-2			ERR072095	P000156	1998				
C-2		C-4	ERR046797	P000176	2006				
C-2		C-4	ERR046798	P000176	2003				
C-2		C-4	ERR046799	P000176	2007	32333	2432515314	434443183	I
C-2		C-4	ERR046822	P000176	2004				
C-2		C-4	ERR046843	P000176	2005	32333	2432515314	434443183	I
C-2		C-4	ERR046908	P000176	2003	32333	2432515314	434443183	I
C-2			ERR039337	P000183	2003	22434	243251a322	423423-52	XV
C-2			ERR039338	P000183	2005	22434	243251a322	423423-52	XV
C-2			ERR046779	P000189	2003	-2235	2542517333		
C-2			ERR046780	P000189	2004	-2235	2542517333		
C-2		C-3 / 4	ERR038294	P000225	2007	32333	2432515314	434443183	I
C-2		C-3 / 4	ERR038295	P000225	2007	32333	2432515314	434443183	I
C-2		C-3 / 4	ERR046785	P000225	2009	32333	2432515314	434443183	I
C-2		C-3 / 4	ERR046795	P000225	2009				

C-2		C-3 / 4	ERR046784	P000226	2008				
C-2		C-3 / 4	ERR046786	P000226	2009				
C-2		C-3 / 4	ERR046787	P000226	2009				
C-2		C-3 / 4	ERR046788	P000226	2009				
C-2		C-3 / 4	ERR046789	P000226	2009				
C-2		C-3 / 4	ERR046790	P000226	2009				
C-2		C-3 / 4	ERR046793	P000226	2008	32333	2432515314	434443183	I
C-2		C-3 / 4	ERR046794	P000226	2008				
C-2		C-3 / 4	ERR046800	P000226	2007	32333	2432515314	434443183	I
C-2		C-3 / 4	ERR046791	P000227	1999	32333	2432515314	434443183	I
C-2		C-3 / 4	ERR046792	P000227	2005				
C-2		C-3 / 4	ERR046911	P000227	2005				
C-2		C-3 / 4	ERR046913	P000227	2005				
C-2			ERR047010	P000259	2008	22434	243251a322		
C-2			ERR047012	P000259	2008				
C-2			ERR046998	P000263	2009				
C-2			ERR047009	P000263	2008	22433	2312513324		
C-2			ERR047011	P000263	2008	22433	2312513324		
C-2			ERR072019	P000263	2008				
C-2			ERR072035	P000263	2008				
C-2			ERR047007	P000267	2007	22232	1332515324		
C-2			ERR047014	P000267	2007				
C-2			ERR072030	P000267	2007				
C-2			ERR072025	P000271	2008				
C-2			ERR072029	P000271	2007	42235	2642516333		
C-2			ERR072026	P000272	2002				
C-2			ERR072040	P000272	2006	42235	-54251-333		
C-2			ERR072031	P000273	2008	-2235	2542517332	54-423372	XXIII
C-2			ERR072032	P000273	2005	-2235	2542517332		
C-2			ERR047013	P000276	2006				
C-2			ERR072037	P000276	2005	42443	2332515324		
C-2			ERR072038	P000276	2005				
C-2			ERR072088	P000320	2008	32333	2432516324		
C-2			ERR072089	P000320	2006	32333	2432516324		
C-2			ERR072090	P000320	2008				
C-3	Household A		ERR039332	P000086	2008	42235	2542516323	542423385	XXII
C-3	Household A		ERR039331	P000087	2008	42235	2542516323	342423385	XXIIa
C-3	Household B		ERR039333	P000116	2007	22235	2542517333	532423384	XXIV
C-3	Household B		ERR039334	P000117	2008	22235	2542517333	332423384	XXIVa
C-3	Household C		ERR039335	P000068	2004	22434	243251a322	4234236-2	XV
C-3	Household C		ERR039336	P000245	2004	22434	243251a322		
C-3	Household D	C-4	ERR039339	P000007	2008	32433	2332514327	223423352	VII
C-3	Household D	C-4	ERR039340	P000108	2008	32433	2332514327	223423352	VII
C-3	Household D	C-4	ERR046930	P000111	2007	32433	2332514327	223423352	VII
C-3	Household E		ERR039341	P000112	2006	22433	2342514322	222423352	X

C-3	Household E		ERR039342	P000218	2005	22433	2342514322	222423352	X
C-3	Household F		ERR046781	P000022	2007	42443	2422515123	415423392	XVI
C-3	Household F		ERR038282	P000102	2007	42443	2422515123	415423392	XVI
C-3	Household F		ERR039343	P000185	2006	42443	2422515123	4254233-2	XVIa
C-3	Household G	C-2 / 4	ERR046783	P000035	2008	32333	2432515315	434443182	Ia*
C-3	Household G	C-4	ERR046782	P000231	2007	32333	2432515315	434443182	Ia*
C-3	Household H	C-4	ERR038281	P000134	2006	32333	2432515314	4344431-3	I
C-3	Household H	C-4	ERR038279	P000135	2004	32333	2432515314	4344-31-3	I
C-3	Household I	C-4	ERR046762	P000131	2008	32433	2312515322	224423542	VIII
C-3	Household I	C-4	ERR038284	P000132	2007	32433	2312515322	224423542	VIII
C-3	Household I	C-4	ERR038283	P000133	2007	32433	2312515322	224423542	VIII
C-3	Household I	C-4	ERR046763	P000140	2007	32433	2312515322	224423542	VIII
C-3	Household J		ERR038287	P000107	2007	42235	2542517333	542423385	XXI
C-3	Household J		ERR038288	P000107	2007	42235	2542517333	342423385	XXIa
C-3	Household J		ERR038280	P000110	2005	42235	2542517333	-424233-5	XXI
C-3	Household K	C-4	ERR038285	P000136	2007	32333	2432515314	434443183	I
C-3	Household K	C-4	ERR038286	P000137	2007	32333	2432515314	434443183	I
C-3	Household L		ERR038290	P000157	2008	12433	1432615324	332413283	XII
C-3	Household L		ERR038293	P000197	2008	12433	1432615324	332413283	XII
C-3	Household M		ERR038291	P000166	2008	33433	2512501322	462554454	XVIII
C-3	Household M		ERR038292	P000188	2008	33433	2512501322	4625544-4	XVIII
C-3	Household N	C-4	ERR038265	P000003	2007	42234	2742511334	432423254	XIX
C-3	Household N	C-4	ERR038266	P000003	2007	42234	2742511334	432423254	XIX
C-3	Household N	C-4	ERR038264	P000004	2007	42234	2742511334	432423254	XIX
C-3	Household O	C-4	ERR038273	P000015	2008	42234	2742511334	432423254	XIX
C-3	Household O	C-4	ERR038271	P000016	2007	42234	2742511334	432423254	XIX
C-3	Household O	C-4	ERR038272	P000017	2008	42234	2742511334	432423254	XIX
C-3	Household O	C-4	ERR038270	P000073	2007	42234	2742511334	432423254	XIX
C-3	Household O	C-4	ERR038269	P000074	2007	42234	2742511334	432423254	XIX
C-3	Household P	C-4	ERR038275	P000115	2005	32333	2432515314	434443183	I
C-3	Household P	C-4	ERR038274	P000125	2003	32333	2432515314	434443183	I
C-3	Household P	C-4	ERR038276	P000125	2003	32333	2432515314	434443183	I
C-3	Household Q		ERR038278	P000021	2003	31333	2232515322	4464433-3	V
C-3	Household Q		ERR038277	P000114	2002	31333	2232515322	446443363	V
C-3	Household R	C-2 / 4	ERR046852	P000095	2004	32333	2432515314	43444318-	Ib
C-3	Household R	C-2 / 4	ERR046853	P000095	2006	32333	2432515314	433443183	Ib
C-3	Household R		ERR046851	P000096	2006	32333	2432515314	433443183	Ib
C-3	Household S		ERR046823	P000207	2004	94265	2432622313	2233237-1	XXVII
C-3	Household S		ERR046824	P000208	2007	94265	2432622313	223323741	XXVII
C-3	Household S		ERR046945	P000209	2009	-4265	2432622313		
C-3	Household S		ERR046850	P000210	2002	94265	2432622313	223323741	XXVII
C-3	Household T	C-4	ERR038296	P000165	2008	32333	2432515314	434443183	I
C-3	Household T	C-4	ERR038297	P000165	2008				
C-3	Household T	C-2 / 4	ERR038294	P000225	2007	32333	2432515314	434443183	I

C-3	Household T	C-2 / 4	ERR038295	P000225	2007	32333	2432515314	434443183	I
C-3	Household T	C-2 / 4	ERR046785	P000225	2009	32333	2432515314	434443183	I
C-3	Household T	C-2 / 4	ERR046795	P000225	2009				
C-3	Household T	C-2 / 4	ERR046784	P000226	2008				
C-3	Household T	C-2 / 4	ERR046786	P000226	2009				
C-3	Household T	C-2 / 4	ERR046787	P000226	2009				
C-3	Household T	C-2 / 4	ERR046788	P000226	2009				
C-3	Household T	C-2 / 4	ERR046789	P000226	2009				
C-3	Household T	C-2 / 4	ERR046790	P000226	2009				
C-3	Household T	C-2 / 4	ERR046793	P000226	2008	32333	2432515314	434443183	I
C-3	Household T	C-2 / 4	ERR046794	P000226	2008				
C-3	Household T	C-2 / 4	ERR046800	P000226	2007	32333	2432515314	434443183	I
C-3	Household T	C-2 / 4	ERR046791	P000227	1999	32333	2432515314	434443183	I
C-3	Household T	C-2 / 4	ERR046792	P000227	2005				
C-3	Household T	C-2 / 4	ERR046911	P000227	2005				
C-3	Household T	C-2 / 4	ERR046913	P000227	2005				
C-3	Household U		ERR072065	P000299	2011	42233	2742511334	432423254	XIXc
C-3	Household U		ERR072077	P000312	2011	42233	2742511334	432423254	XIXc
C-3	Household U		ERR072080	P000315	2011	42233	2742511334	432423254	XIXc
C-3	Household V		ERR046921	P000023	2007				XIX
C-3	Household V	C-4	ERR040106	P000240	2008	42234	2742511334	432423254	XIX
C-3	Household W	C-2 /4	ERR040087	P000026	2006	-2234	2742511334	432422244	XIXb*
C-3	Household W	C-2 /4	ERR046770	P000026	2008	-2234	2742511334		
C-3	Household W	C-2 /4	ERR046940	P000026	2007				
C-3	Household W	C-2 /4	ERR046942	P000026	2007				
C-3	Household W	C-2 / 4	ERR038298	P000027	2006	42234	2742511334	432422244	XIXb*
C-3	Household W	C-2 / 4	ERR046769	P000027	2008	-2234	2742511334	432422244	XIXb*
C-3	Household X	C-4	ERR046950	P000039	2008	32433	2432515324	442443151	IIa*
C-3	Household X	C-4	ERR039328	P000040	2007	32433	2432515324	442443151	IIa*
C-3	Household Y	C-4	ERR046733	P000145	2002	32433	2432515324	443443153	II
C-3	Household Y	C-4	ERR046738	P000146	1999	32433	2432515324	443443153	II
C-3	Household Y	C-4	ERR040109	P000147	2002	32433	2432515324	443443153	II
C-4	Cluster 1	C-3	ERR039339	P000007	2008	32433	2332514327	223423352	VII
C-4	Cluster 1	C-2	ERR046929	P000048	2009	32433	2332514327	223423352	VII
C-4	Cluster 1	C-2	ERR046948	P000048	2008	32433	2332514327	223423352	VII
C-4	Cluster 1		ERR046952	P000099	2008	32433	2332514327	2-3423352	VII
C-4	Cluster 1	C-3	ERR039340	P000108	2008	32433	2332514327	223423352	VII
C-4	Cluster 1		ERR046932	P000109	2008	32433	2332514327	223423352	VII
C-4	Cluster 1	C-3	ERR046930	P000111	2007	32433	2332514327	223423352	VII
C-4	Cluster 1		ERR046953	P000154	2008	32433	2332514327	223423352	VII
C-4	Cluster 1		ERR046951	P000186	2008	32433	2332514327	223423352	VII
C-4	Cluster 2		ERR046957	P000036	2009	32333	2512515324		
C-4	Cluster 2		ERR046959	P000070	2009	32333	2512515324		

C-4	Cluster 2		ERR046958	P000129	2009	32333	2512515324		
C-4	Cluster 2		ERR046974	P000169	2009	32333	2512515324		
C-4	Cluster 2		ERR046972	P000194	2009	32333	2512515324	23443-363	VI
C-4	Cluster 2		ERR046993	P000250	2010	32333	2512515324	234433363	VI
C-4	Cluster 2		ERR046990	P000253	2009	32333	2512515324		
C-4	Cluster 2		ERR046994	P000254	2002	32333	2512515324		
C-4	Cluster 2		ERR046992	P000256	2010	32333	2512515324	234433363	VI
C-4	Cluster 3		ERR046970	P000123	2008	42234	2742511324	432423254	XIXa
C-4	Cluster 3	C-1	ERR025833	P000149	2006	42234	2742511324	432423254	XIXa
C-4	Cluster 3		ERR046969	P000198	2010	42234	2742511324	432423254	XIXa
C-4	Cluster 3		ERR046965	P000224	2011	42234	2742511324	432423254	XIXa
C-4	Cluster 3		ERR046968	P000248	2008	42234	2742511324	432423254	XIXa
C-4	Cluster 3		ERR072094	P000324	2009	42234	2742511324	432423254	XIXa
C-4	Cluster 4	C-1	ERR025837	P000002	2007	32433	2312515322	-24423542	VIII
C-4	Cluster 4	C-1	ERR025838	P000002	2007				
C-4	Cluster 4		ERR046920	P000019	2009	32433	2312515322	224423542	VIII
C-4	Cluster 4		ERR046755	P000028	2007	32433	2312515322	22-42354-	VIII
C-4	Cluster 4		ERR040101	P000029	2007	32433	2312515322	024423542	VIIIa
C-4	Cluster 4		ERR046926	P000032	2009	32433	2312515322	224423542	VIII
C-4	Cluster 4	C-1	ERR046878	P000034	2006	32433	2312515322		
C-4	Cluster 4	C-1	ERR046879	P000034	2006				
C-4	Cluster 4		ERR040103	P000038	2006	32433	2312515322		
C-4	Cluster 4		ERR040100	P000044	2006	32433	2312515322		
C-4	Cluster 4		ERR046756	P000052	2008	32433	2312515322	224423542	VIII
C-4	Cluster 4		ERR040099	P000058	2006	32433	2312515322	224423542	VIII
C-4	Cluster 4		ERR046925	P000078	2005	32433	2312515322		
C-4	Cluster 4		ERR046751	P000079	2005	32433	2312515322		
C-4	Cluster 4		ERR040102	P000080	2007	32433	2312515322	224423542	VIII
C-4	Cluster 4		ERR046758	P000080	2008	32433	2312515322	2-4423542	VIII
C-4	Cluster 4	C-1	ERR038254	P000101	2006	32433	2312515322		
C-4	Cluster 4	C-1	ERR038255	P000101	2006				
C-4	Cluster 4		ERR046759	P000103	2008	32433	2312515322	224423542	VIII
C-4	Cluster 4		ERR040104	P000104	2005	32433	2312515322		
C-4	Cluster 4		ERR046760	P000120	2008	32433	2312515322	224423542	VIII
C-4	Cluster 4		ERR046761	P000121	2008	32433	2312515322	224423542	VIII
C-4	Cluster 4		ERR046754	P000122	2006	32433	2312515322		
C-4	Cluster 4	C-1	ERR046876	P000124	2007	33433	2312515322		
C-4	Cluster 4	C-1	ERR046877	P000124	2007	33433	2312515322		
C-4	Cluster 4		ERR040096	P000130	2006	32433	2312515322		
C-4	Cluster 4	C-3	ERR046762	P000131	2008	32433	2312515322	224423542	VIII
C-4	Cluster 4	C-3	ERR038284	P000132	2007	32433	2312515322	224423542	VIII
C-4	Cluster 4	C-3	ERR038283	P000133	2007	32433	2312515322	224423542	VIII
C-4	Cluster 4	C-3	ERR046763	P000140	2007	32433	2312515322	224423542	VIII
C-4	Cluster 4		ERR046764	P000160	2008	32433	2312515322	224423542	VIII
C-4	Cluster 4		ERR046753	P000163	2005	32433	2312515322		

C-4	Cluster 4		ERR046765	P000173	2008	32433	2312515322	224423542	VIII
C-4	Cluster 4		ERR040094	P000177	2007	32433	2312515322	224423542	VIII
C-4	Cluster 4		ERR040105	P000187	2003	32433	2312515322		
C-4	Cluster 4		ERR046820	P000187	2003				
C-4	Cluster 4		ERR040098	P000191	2006	32433	2312515322	224423-42	VIII
C-4	Cluster 4		ERR040095	P000215	2006	32433	2312515322		
C-4	Cluster 4		ERR040097	P000215	2006	32433	2312515322	224423542	VIII
C-4	Cluster 4		ERR046766	P000222	2005	32433	2312515322		
C-4	Cluster 4		ERR046767	P000223	2008	32433	2312515322	22--235-2	VIII
C-4	Cluster 4		ERR046752	P000235	2005	32433	2312515322		
C-4	Cluster 4		ERR046768	P000243	2008	32433	2312515322	224423542	VIII
C-4	Cluster 4		ERR046928	P000244	2009	32433	2312515322	224423542	VIII
C-4	Cluster 4		ERR046927	P000246	2005	32433	2312515322		
C-4	Cluster 4		ERR072041	P000277	2009	32433	2312515322	224423542	VIII
C-4	Cluster 4		ERR072042	P000278	2009	32433	2312515322	224423542	VIII
C-4	Cluster 4		ERR072044	P000280	2011	32433	2312515322	224423542	VIII
C-4	Cluster 4		ERR072045	P000281	2010	32433	2312515322	224423542	VIII
C-4	Cluster 4		ERR072046	P000282	2009	32433	2312515322	224423542	VIII
C-4	Cluster 4		ERR072047	P000283	2009	32433	2312515322	224423542	VIII
C-4	Cluster 4		ERR072048	P000284	2009	32433	2312515322	224423542	VIII
C-4	Cluster 4		ERR072050	P000286	2009	32433	2312515322	224423542	VIII
C-4	Cluster 4		ERR072051	P000287	2010	32433	2312515322	224423542	VIII
C-4	Cluster 5		ERR046975	P000077	2002	32433	2432515323		
C-4	Cluster 5		ERR046946	P000182	2010	32433	2432515323	241433273	III
C-4	Cluster 5		ERR046962	P000203	2009	32433	2432515323		
C-4	Cluster 5		ERR046739	P000230	2002	32433	2432515323	241433273	III
C-4	Cluster 5		ERR046991	P000251	2005	32433	2432515323		
C-4	Cluster 5		ERR046988	P000255	2007	32433	2432515323		
C-4	Cluster 5		ERR072020	P000265	2005	32433	2432515323		
C-4	Cluster 5		ERR072034	P000274	1998	32433	2432515323		
C-4	Cluster 5		ERR072036	P000275	1999	32433	2432515323		
C-4	Cluster 6	C-3	ERR046950	P000039	2008	32433	2432515324	442443151	IIa*
C-4	Cluster 6	C-3	ERR039328	P000040	2007	32433	2432515324	442443151	IIa*
C-4	Cluster 6		ERR046924	P000041	2009	32433	2432515324	443443153	II
C-4	Cluster 6		ERR046745	P000046	2008	32433	2432515324	443443153	II
C-4	Cluster 6		ERR046737	P000050	2007	32433	2432515324	443443153	II
C-4	Cluster 6		ERR046960	P000057	1998	32433	2432515324		
C-4	Cluster 6		ERR046741	P000060	2005	32433	2432515324	443443153	II
C-4	Cluster 6		ERR046744	P000067	2000	32433	2432515324	443443153	II
C-4	Cluster 6	C-2	ERR046825	P000071	2007				
C-4	Cluster 6	C-2	ERR046937	P000071	2006				
C-4	Cluster 6	C-2	ERR046943	P000071	2006				
C-4	Cluster 6	C-2	ERR046986	P000071	2004				
C-4	Cluster 6		ERR046734	P000090	2006	32433	2432515324		
C-4	Cluster 6		ERR046971	P000105	2009	32433	2432515324	443443153	II

C-4	Cluster 6		ERR039329	P000113	2007	32433	2432515324	443443153	II
C-4	Cluster 6		ERR046841	P000119	2007	32433	2432515324	442443151	IIa*
C-4	Cluster 6		ERR046964	P000139	2010	32433	2432515324	443443153	II
C-4	Cluster 6		ERR046735	P000142	2007	32433	2432515324	44-443153	II
C-4	Cluster 6		ERR046746	P000143	2008	32433	2432515324	443443153	II
C-4	Cluster 6	C-3	ERR046733	P000145	2002	32433	2432515324	443443153	II
C-4	Cluster 6	C-3	ERR046738	P000146	1999	32433	2432515324	443443153	II
C-4	Cluster 6	C-3	ERR040109	P000147	2002	32433	2432515324	443443153	II
C-4	Cluster 6		ERR046923	P000151	2010	32433	2432515324	443443153	II
C-4	Cluster 6		ERR046840	P000199	2007	32433	2432515324	443443153	II
C-4	Cluster 6		ERR039327	P000205	2007	32433	2432515324	4434431--	II
C-4	Cluster 6		ERR046819	P000212	2009	32433	2432515324	442443151	IIa*
C-4	Cluster 6		ERR046736	P000229	2005	32433	2432515324	443443153	II
C-4	Cluster 6		ERR046743	P000237	2000	32433	2432515324	443443153	II
C-4	Cluster 6		ERR046842	P000241	2008	32433	2432515324	443443153	II
C-4	Cluster 7	C-2 / 3	ERR040087	P000026	2006	-2234	2742511334	432422244	XIXb*
C-4	Cluster 7	C-2 / 3	ERR046770	P000026	2008	-2234	2742511334		
C-4	Cluster 7	C-2 / 3	ERR046940	P000026	2007				
C-4	Cluster 7	C-2 / 3	ERR046942	P000026	2007				
C-4	Cluster 7	C-2 / 3	ERR038298	P000027	2006	42234	2742511334	432422244	XIXb*
C-4	Cluster 7	C-2 / 3	ERR046769	P000027	2008	-2234	2742511334	432422244	XIXb*
C-4	Cluster 7		ERR040088	P000037	2005	-2234	2742511334	432422254	XIXb
C-4	Cluster 7		ERR046771	P000066	2008	-2234	2742511334	432422254	XIXb
C-4	Cluster 7	C-2	ERR039323	P000076	2002	-2234	2742511334	432422254	XIXb
C-4	Cluster 7	C-2	ERR046729	P000076	2004	-2234	2742511334	432422254	XIXb
C-4	Cluster 7	C-2	ERR046772	P000076	2004	-2234	2742511334	432422254	XIXb
C-4	Cluster 7	C-2	ERR046914	P000076	2005	-2234	2742511334	4324222--	XIXb
C-4	Cluster 7		ERR040108	P000174	2004	-2234	2742511334	432422254	XIXb
C-4	Cluster 7		ERR040090	P000175	2007	-2234	2742511334	432422254	XIXb
C-4	Cluster 7		ERR046773	P000211	2007	-2234	2742511334	432422254	XIXb
C-4	Cluster 7		ERR192249	P000334	2011	'-2234	2742-11334	432422254	XIXb
C-4	Cluster 7		ERR192250	P000335	2011	'-2234	2742511334	432422254	XIXb
C-4	Cluster 8		ERR046938	P000005	2010	42435	2332517333	34-443584	XXVI
C-4	Cluster 8		ERR046949	P000020	2010	42435	2332517333	346443584	XXVI
C-4	Cluster 8		ERR046966	P000051	2010	42435	2332517333	346443584	XXVI
C-4	Cluster 8		ERR046947	P000127	2010	42435	2332517333	346443584	XXVI
C-4	Cluster 8		ERR046967	P000153	2010	42435	2332517333	346443584	XXVI
C-4	Cluster 8		ERR046963	P000214	2010	42435	2332517333	346443584	XXVI
C-4	Cluster 9	C-2 / 3	ERR046783	P000035	2008	32333	2432515315	434443182	Ia*
C-4	Cluster 9	C-2	ERR038253	P000089	2006	32333	2432515314	434443183	I
C-4	Cluster 9	C-2	ERR038256	P000089	2007	32333	2432515314	434443183	I
C-4	Cluster 9	C-2	ERR038257	P000089	2008				
C-4	Cluster 9	C-2	ERR039346	P000089	2005	32333	2422515314		
C-4	Cluster 9	C-2	ERR046904	P000089	2008				
C-4	Cluster 9	C-2	ERR046910	P000089	2006				

C-4	Cluster 9	C-2	ERR046919	P000089	2006				
C-4	Cluster 9	C-2 / 3	ERR046852	P000095	2004	32333	2432515314	43444318-	Ib
C-4	Cluster 9	C-2 / 3	ERR046853	P000095	2006	32333	2432515314	433443183	Ib
C-4	Cluster 9	C-3	ERR038275	P000115	2005	32333	2432515314	434443183	I
C-4	Cluster 9	C-3	ERR038274	P000125	2003	32333	2432515314	434443183	I
C-4	Cluster 9	C-3	ERR038276	P000125	2003	32333	2432515314	434443183	I
C-4	Cluster 9	C-3	ERR038281	P000134	2006	32333	2432515314	4344431-3	I
C-4	Cluster 9	C-3	ERR038279	P000135	2004	32333	2432515314	4344-31-3	I
C-4	Cluster 9	C-3	ERR038285	P000136	2007	32333	2432515314	434443183	I
C-4	Cluster 9	C-3	ERR038286	P000137	2007	32333	2432515314	434443183	I
C-4	Cluster 9	C-3	ERR038296	P000165	2008	32333	2432515314	434443183	I
C-4	Cluster 9	C-3	ERR038297	P000165	2008				
C-4	Cluster 9	C-2	ERR046797	P000176	2006				
C-4	Cluster 9	C-2	ERR046798	P000176	2003				
C-4	Cluster 9	C-2	ERR046799	P000176	2007	32333	2432515314	434443183	I
C-4	Cluster 9	C-2	ERR046822	P000176	2004				
C-4	Cluster 9	C-2	ERR046843	P000176	2005	32333	2432515314	434443183	I
C-4	Cluster 9	C-2	ERR046908	P000176	2003	32333	2432515314	434443183	I
C-4	Cluster 9		ERR046917	P000179	2003	32333	2432515314	434443183	I
C-4	Cluster 9	C-2 / 3	ERR038294	P000225	2007	32333	2432515314	434443183	I
C-4	Cluster 9	C-2 / 3	ERR038295	P000225	2007	32333	2432515314	434443183	I
C-4	Cluster 9	C-2 / 3	ERR046785	P000225	2009	32333	2432515314	434443183	I
C-4	Cluster 9	C-2 / 3	ERR046795	P000225	2009				
C-4	Cluster 9	C-2 / 3	ERR046784	P000226	2008				
C-4	Cluster 9	C-2 / 3	ERR046786	P000226	2009				
C-4	Cluster 9	C-2 / 3	ERR046787	P000226	2009				
C-4	Cluster 9	C-2 / 3	ERR046788	P000226	2009				
C-4	Cluster 9	C-2 / 3	ERR046789	P000226	2009				
C-4	Cluster 9	C-2 / 3	ERR046790	P000226	2009				
C-4	Cluster 9	C-2 / 3	ERR046793	P000226	2008	32333	2432515314	434443183	I
C-4	Cluster 9	C-2 / 3	ERR046794	P000226	2008				
C-4	Cluster 9	C-2 / 3	ERR046800	P000226	2007	32333	2432515314	434443183	I
C-4	Cluster 9	C-2 / 3	ERR046791	P000227	1999	32333	2432515314	434443183	I
C-4	Cluster 9	C-2 / 3	ERR046792	P000227	2005				
C-4	Cluster 9	C-2 / 3	ERR046911	P000227	2005				
C-4	Cluster 9	C-2 / 3	ERR046913	P000227	2005				
C-4	Cluster 9	C-3	ERR046782	P000231	2007	32333	2432515315	434443182	Ia*
C-4	Cluster 9		ERR072087	P000319	2007	32333	2432515314	434443183	I
C-4	Cluster 9		ERR072096	P000325	2006	32333	2432515314	434443183	I
C-4	Cluster 10		ERR046847	P000001	2007	42234	2742511334	4324232-4	XIX
C-4	Cluster 10	C-3	ERR038265	P000003	2007	42234	2742511334	432423254	XIX
C-4	Cluster 10	C-3	ERR038266	P000003	2007	42234	2742511334	432423254	XIX
C-4	Cluster 10	C-3	ERR038264	P000004	2007	42234	2742511334	432423254	XIX
C-4	Cluster 10		ERR038300	P000006	2006	42234	2742511334	432423254	XIX
C-4	Cluster 10	C-3	ERR038273	P000015	2008	42234	2742511334	432423254	XIX

C-4	Cluster 10	C-3	ERR038271	P000016	2007	42234	2742511334	432423254	XIX
C-4	Cluster 10	C-3	ERR038272	P000017	2008	42234	2742511334	432423254	XIX
C-4	Cluster 10		ERR046854	P000018	2000	42234	2-42511334	432423254	XIX
C-4	Cluster 10	C-3	ERR046921	P000023	2007	42234	2742511334	43242325-	XIX
C-4	Cluster 10		ERR046775	P000024	2008	42234	2742511334	432423254	XIX
C-4	Cluster 10		ERR046845	P000063	2008	42234	2742511334	432423254	XIX
C-4	Cluster 10		ERR038299	P000069	2006	42234	2742511334	432423254	XIX
C-4	Cluster 10	C-3	ERR038270	P000073	2007	42234	2742511334	4324232-4	XIX
C-4	Cluster 10	C-3	ERR038269	P000074	2007	42234	2742511334	432423254	XIX
C-4	Cluster 10	C-1	ERR046982	P000081	2007	4223-	2742511334	432423254	XIX
C-4	Cluster 10		ERR040091	P000084	2007	42234	2742511334	432423254	XIX
C-4	Cluster 10		ERR040107	P000098	2005	42234	2742511334	432423254	XIX
C-4	Cluster 10		ERR040089	P000158	2007	42234	2742511334	432423254	XIX
C-4	Cluster 10		ERR040086	P000161	2006	42234	2742511334	432423254	XIX
C-4	Cluster 10	C-1	ERR046866	P000167	2007	42234	2742511334		
C-4	Cluster 10	C-1	ERR046867	P000167	2007	42234	2742511334		
C-4	Cluster 10		ERR040093	P000239	2003	42234	2742511334	432423254	XIX
C-4	Cluster 10	C-3	ERR040106	P000240	2008	42234	2742511334	432423254	XIX
C-4	Cluster 10	C-1	ERR046860	P000859	2008	42234	2742511334		
C-4	Cluster 10	C-1	ERR046861	P000859	2008	42234	2742511334		
C-4	Cluster 11		ERR046747	P000047	2006	75553	2222415322	234323241	XXX
C-4	Cluster 11		ERR046748	P000144	2005	75553	2222415322	---------	
C-4	Cluster 11		ERR046954	P000168	2005	75553	2222415322	234323241	XXX
C-4	Cluster 11		ERR046961	P000178	2010	75553	2222415322	234323241	XXX
C-4	Cluster 11		ERR046749	P000181	2008	75553	2222415322	234323241	XXX
C-4	Cluster 11		ERR046989	P000238	2005	75553	2222415322	234323241	XXX

Figures S3 and S4 show maximum-likelihood (ML) trees illustrating the diversity within individuals and between epidemiologically related individuals. S5 shows the ML trees for the community clusters. Patients are presented in nodes, indexed by patient numbers, together with the calendar year each sample was taken. Yellow nodes containing more than one patient number represent multiple patients with WGS that are 0 SNPs apart. Black nodes divide branches to mark SNP distances. Dashed lines show larger SNP distances (not to scale). In S5, arrows indicate the next closest isolate in the sequenced collection.

<u>S3.</u> Individuals with ≥2 sputum isolates over time (at least 6 months apart):

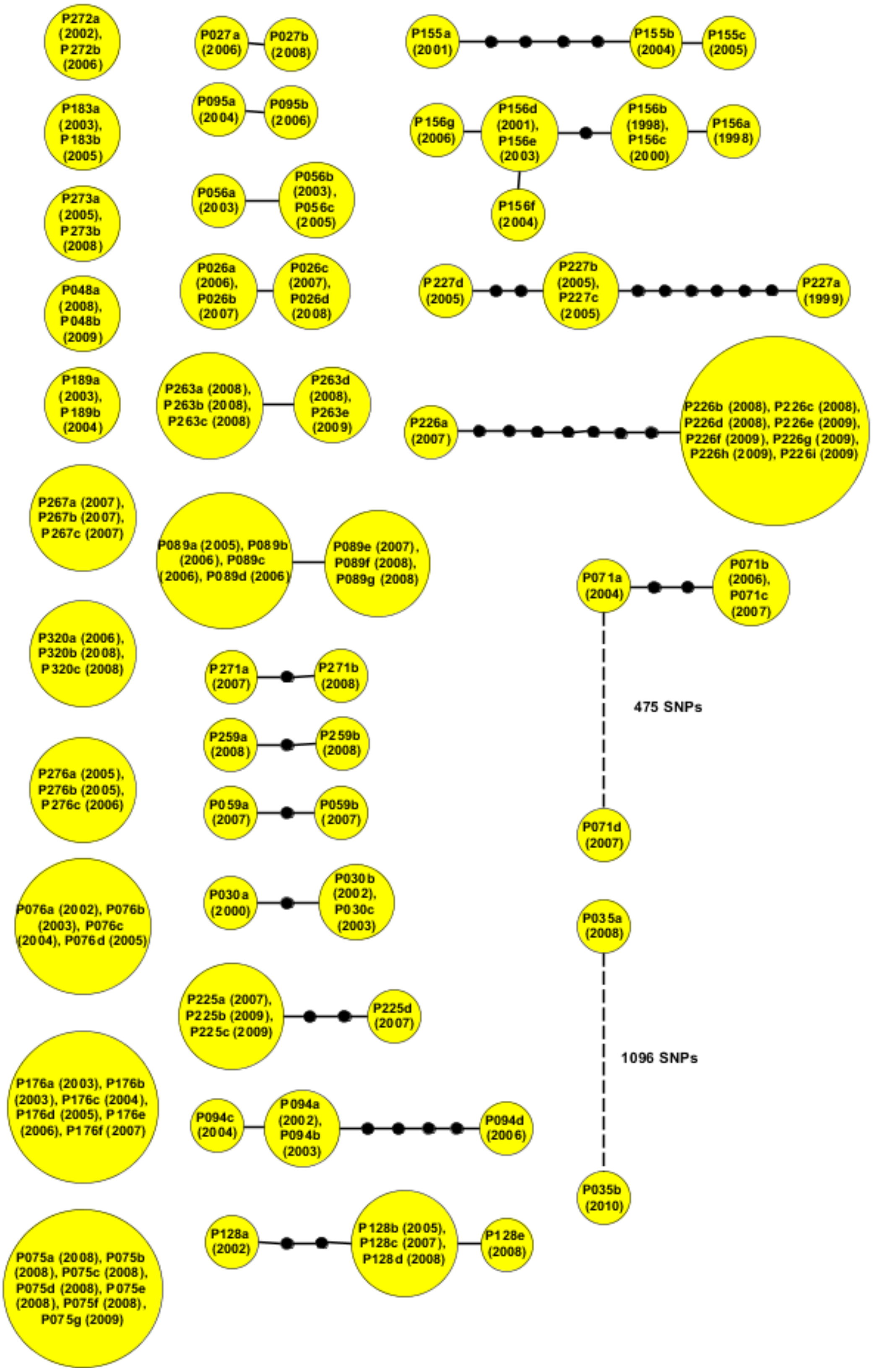

S4. Families / household clusters:

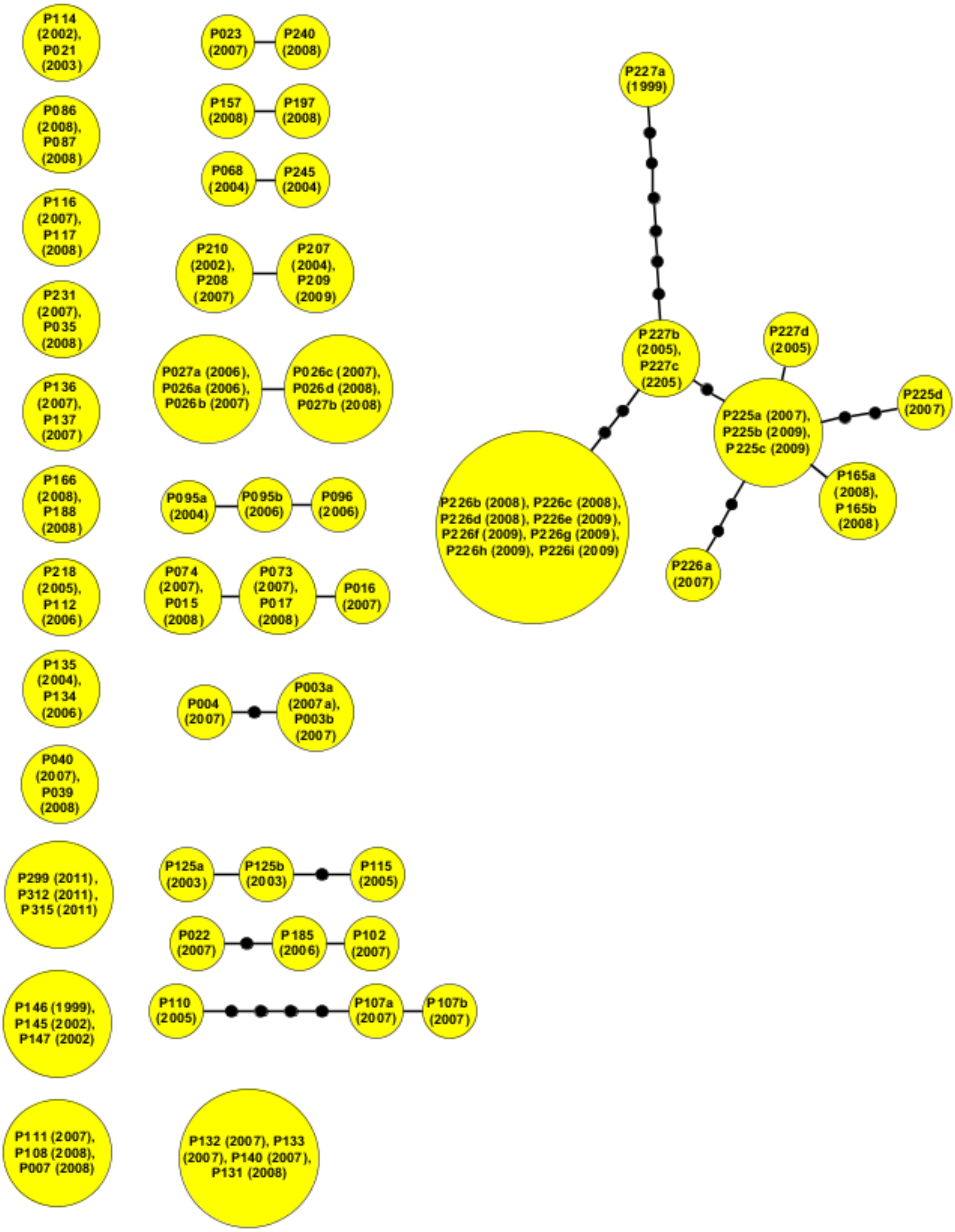

S5. MIRU-VNTR based community clusters:

Cluster 1

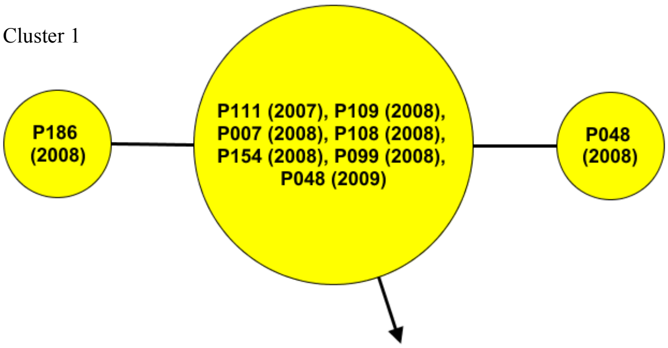

Cluster 2

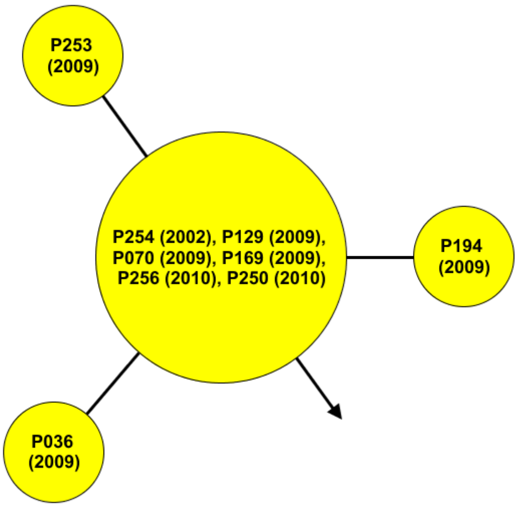

Cluster 3

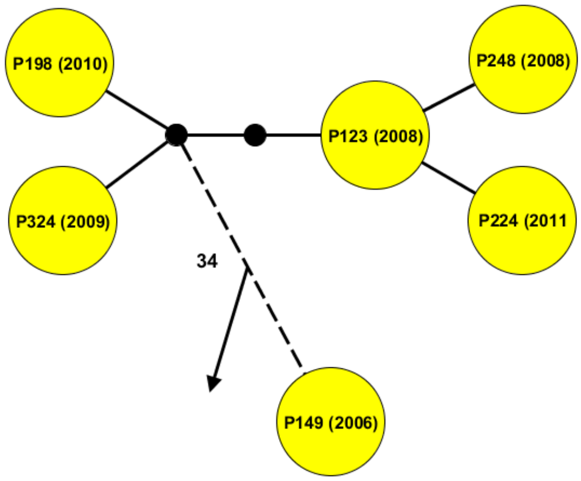

Cluster 4

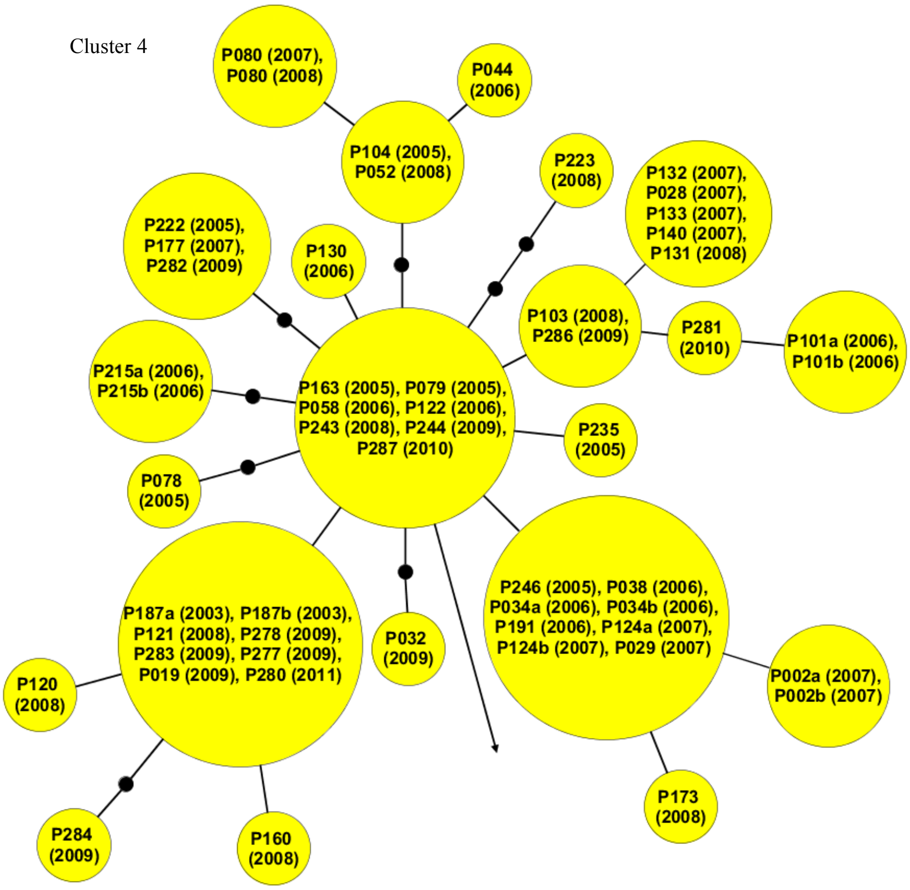

Cluster 5

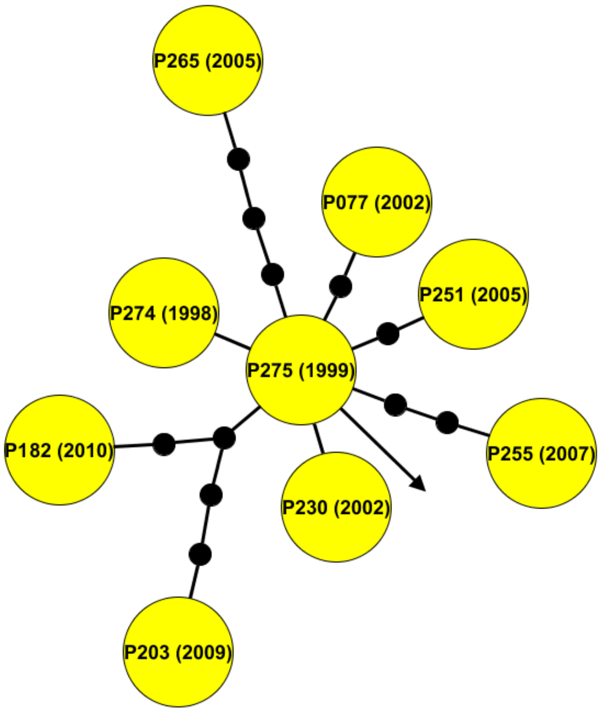

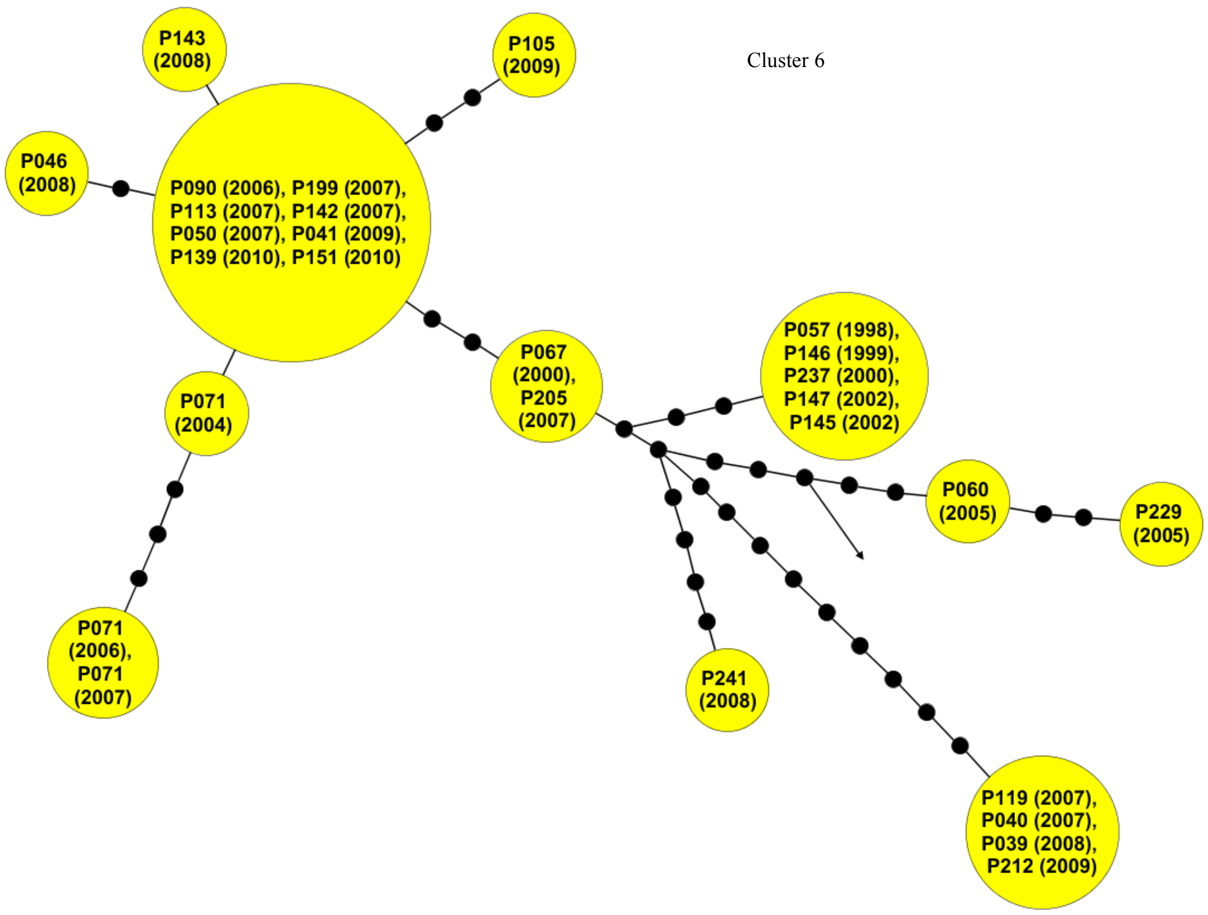
Cluster 6
P143 (2008)
P105 (2009)
P046 (2008)
P090 (2006), P199 (2007), P113 (2007), P142 (2007), P050 (2007), P041 (2009), P139 (2010), P151 (2010)
P071 (2004)
P071 (2006), P071 (2007)
P067 (2000), P205 (2007)
P057 (1998), P146 (1999), P237 (2000), P147 (2002), P145 (2002)
P060 (2005)
P229 (2005)
P241 (2008)
P119 (2007), P040 (2007), P039 (2008), P212 (2009)

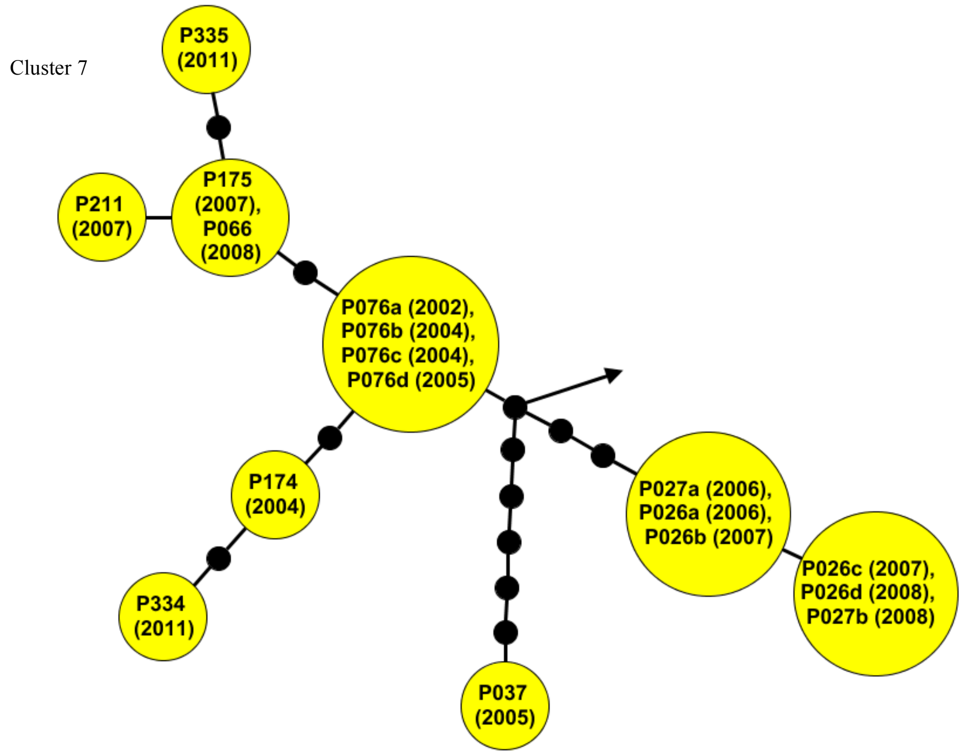
Cluster 7
P335 (2011)
P211 (2007)
P175 (2007), P066 (2008)
P076a (2002), P076b (2004), P076c (2004), P076d (2005)
P174 (2004)
P334 (2011)
P027a (2006), P026a (2006), P026b (2007)
P026c (2007), P026d (2008), P027b (2008)
P037 (2005)

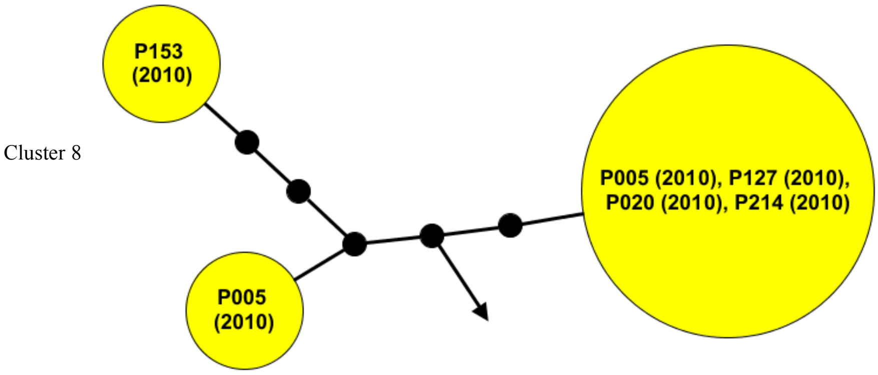
Cluster 8
P153 (2010)
P005 (2010), P127 (2010), P020 (2010), P214 (2010)
P005 (2010)

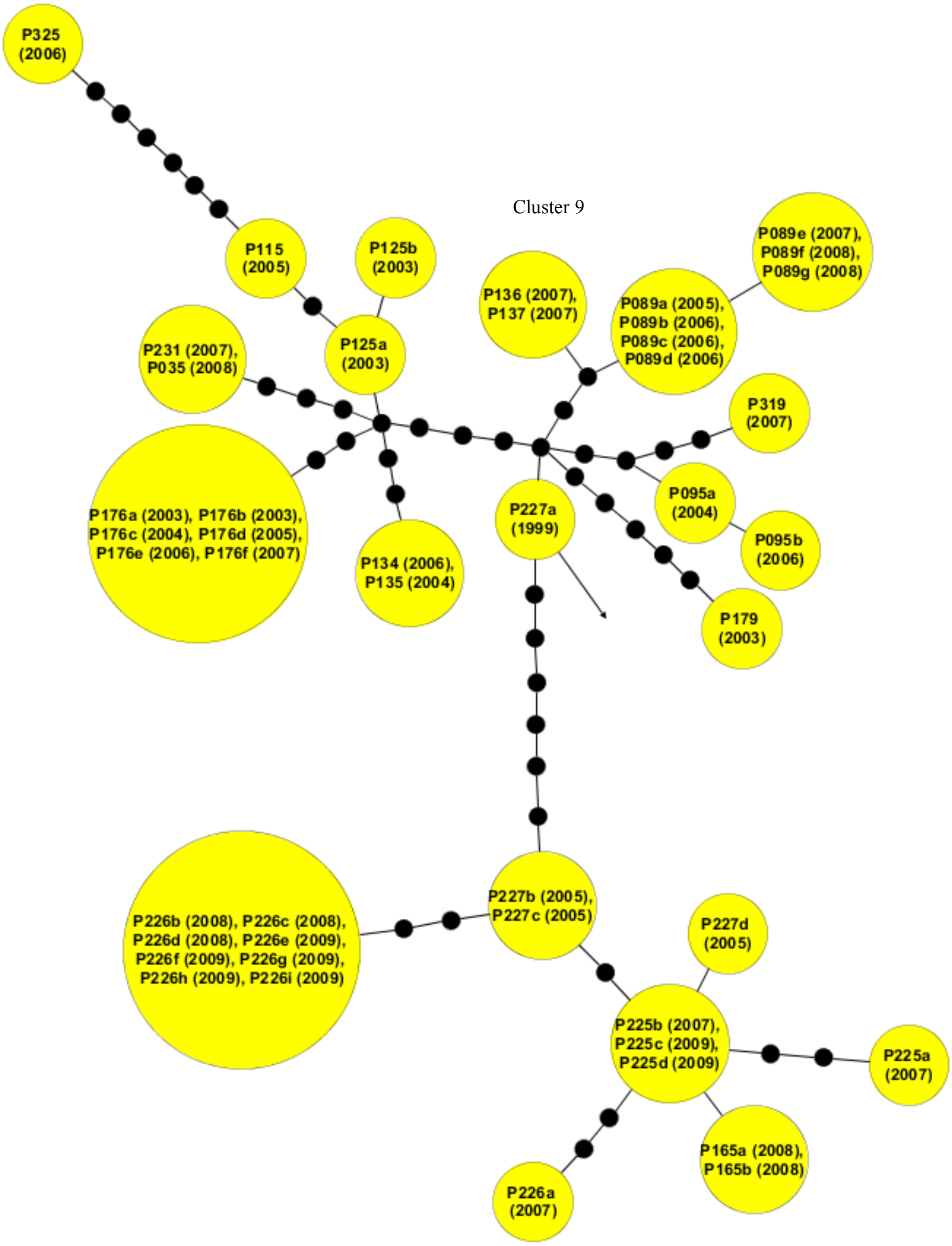
Cluster 9
P325 (2006)
P115 (2005)
P125b (2003)
P125a (2003)
P231 (2007), P035 (2008)
P176a (2003), P176b (2003), P176c (2004), P176d (2005), P176e (2006), P176f (2007)
P134 (2006), P135 (2004)
P136 (2007), P137 (2007)
P089a (2005), P089b (2006), P089c (2006), P089d (2006)
P089e (2007), P089f (2008), P089g (2008)
P319 (2007)
P095a (2004)
P095b (2006)
P179 (2003)
P227a (1999)
P226b (2008), P226c (2008), P226d (2008), P226e (2009), P226f (2009), P226g (2009), P226h (2009), P226i (2009)
P227b (2005), P227c (2005)
P227d (2005)
P225b (2007), P225c (2009), P225d (2009)
P225a (2007)
P165a (2008), P165b (2008)
P226a (2007)

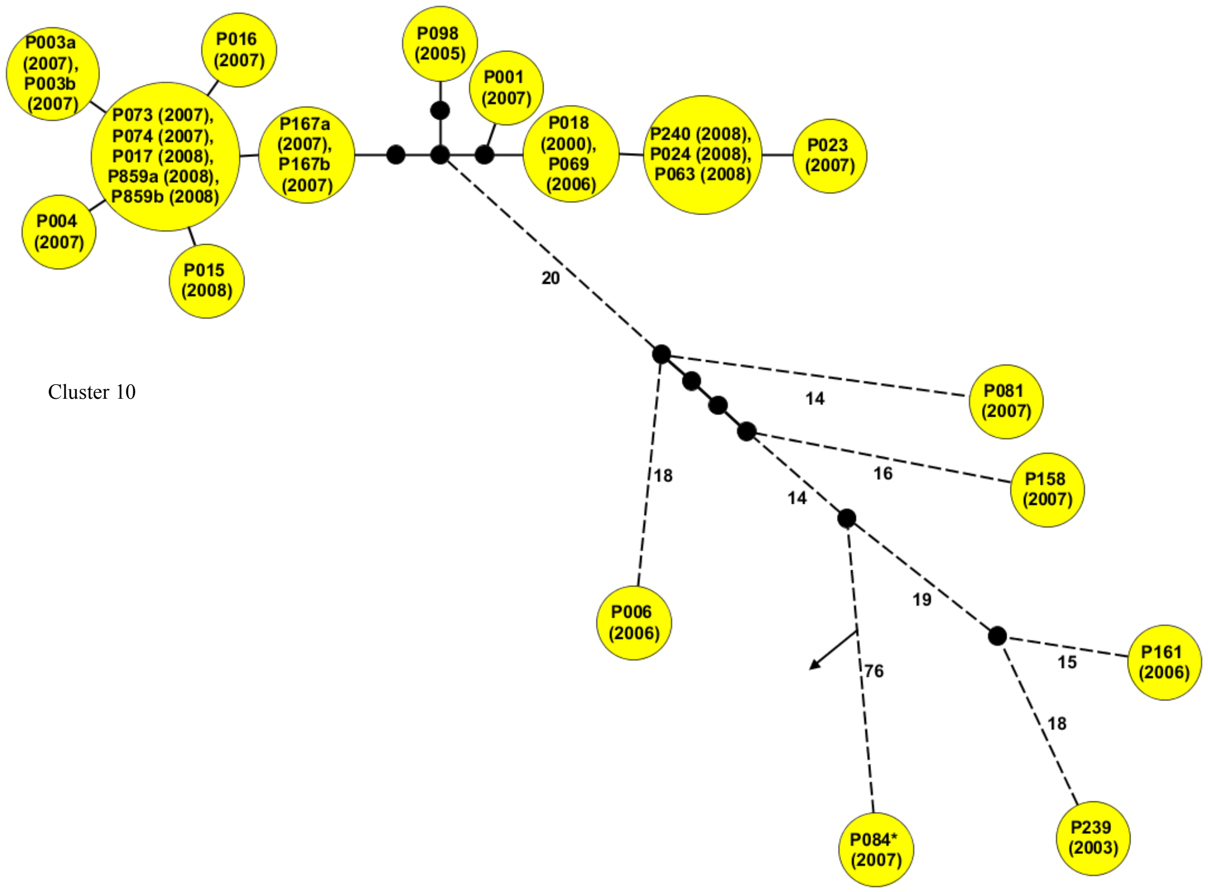

Cluster 10

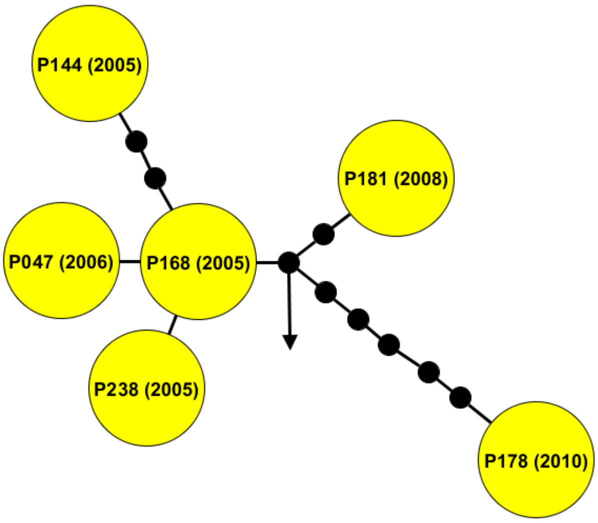

Cluster 11

S6. Summary of colour-coded epidemiological links within 11 community clusters: green = known link; orange = possible link; red = no known link. Each patient is depicted by a study number within a red node. Edges linking the nodes are annotated by the minimum SNPs/months between patient isolates.

The networks are an attempt to summarise the links between patients in each cluster in a way that maximises epidemiological and genetic proximity. To avoid double counting we restricted the number of connecting edges to the number of nodes minus one. Methods used to produce network diagrams were as follows: For each cluster we started with the first patient to be diagnosed. We sought to draw a link (an 'edge') to another patient with a 'known' epidemiological link. If there was >1 patient to choose from, we chose the patient with an isolate closest in SNPs, and where this failed to identify a unique edge, we chose the patient closest in time (as judged by date of isolation of sample). If no 'known' epidemiological link existed, we sought 'possible' linkage before 'no known' linkage, in each instance prioritising by SNPs and time as described. The second edge and all subsequent edges were determined by the same rules until the network was complete.

Cluster 1 Cluster 2 Cluster 3

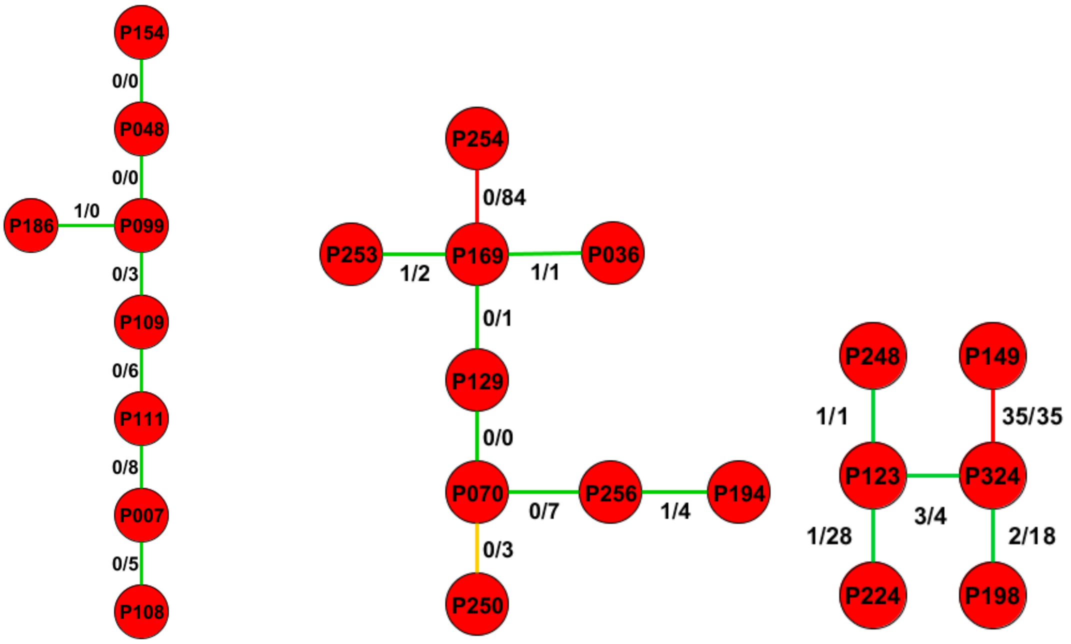

Cluster 4

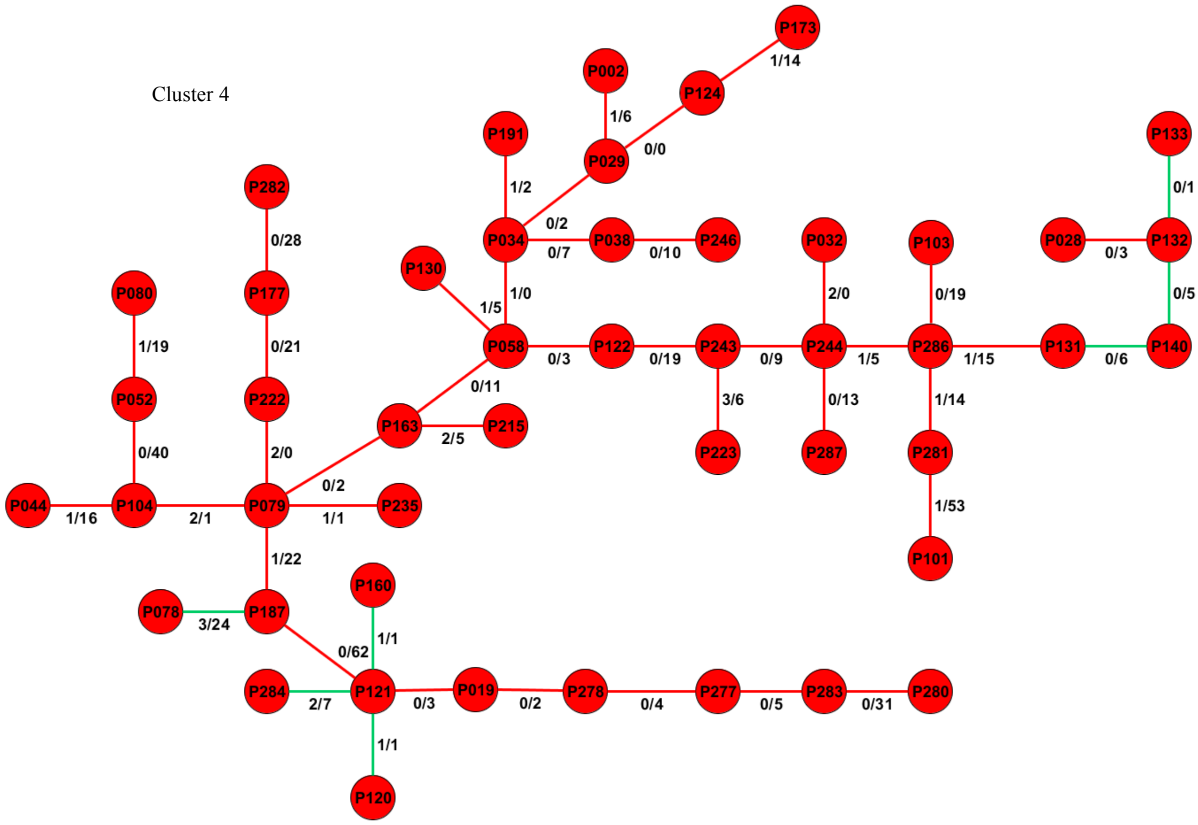

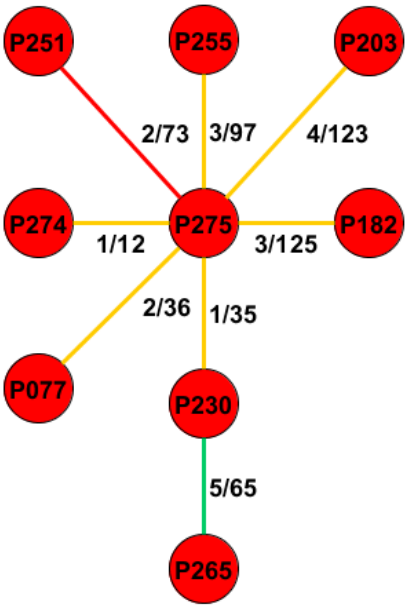

Cluster 5

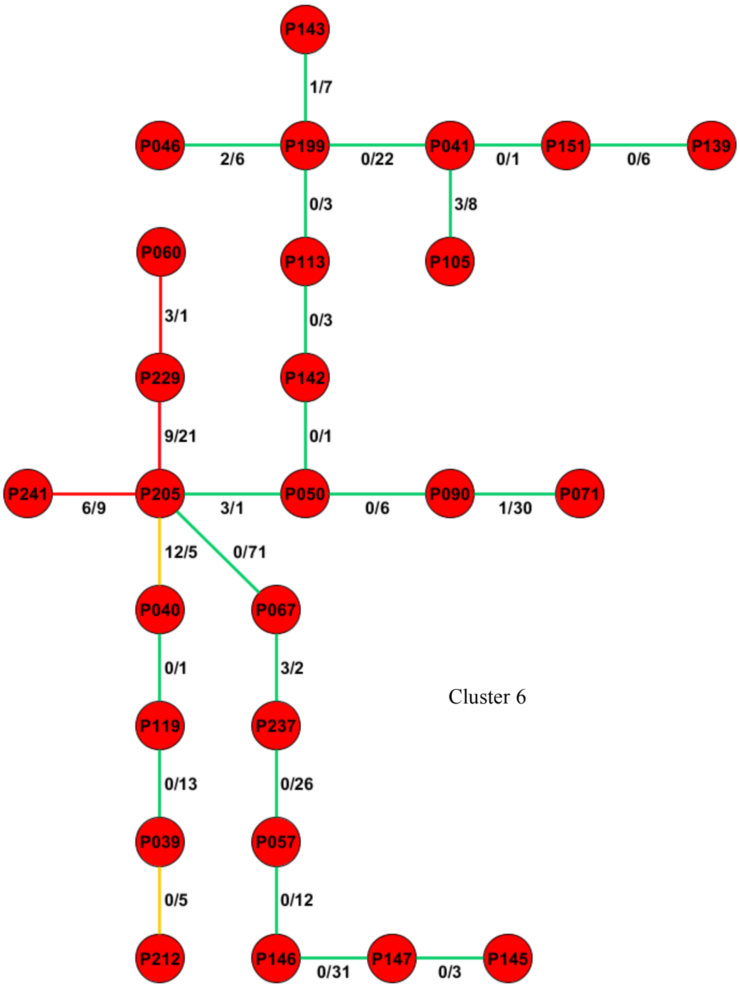

Cluster 6

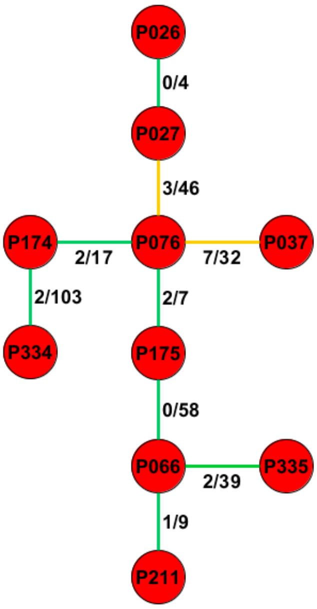

Cluster 7

Cluster 8

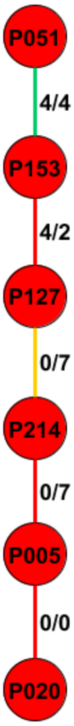

Cluster 9

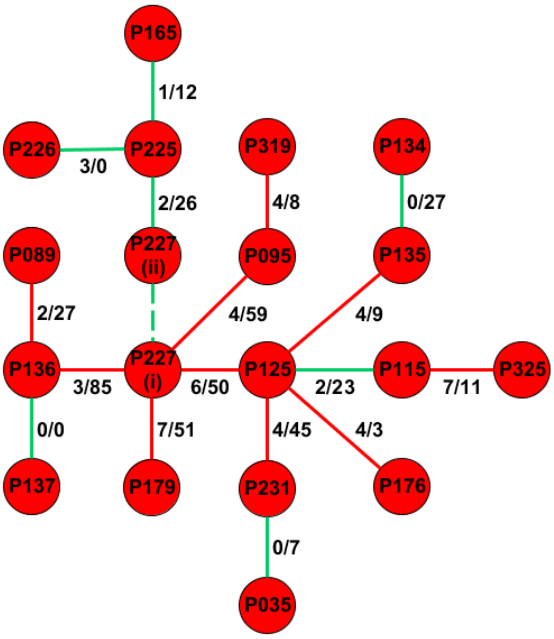

Cluster 10

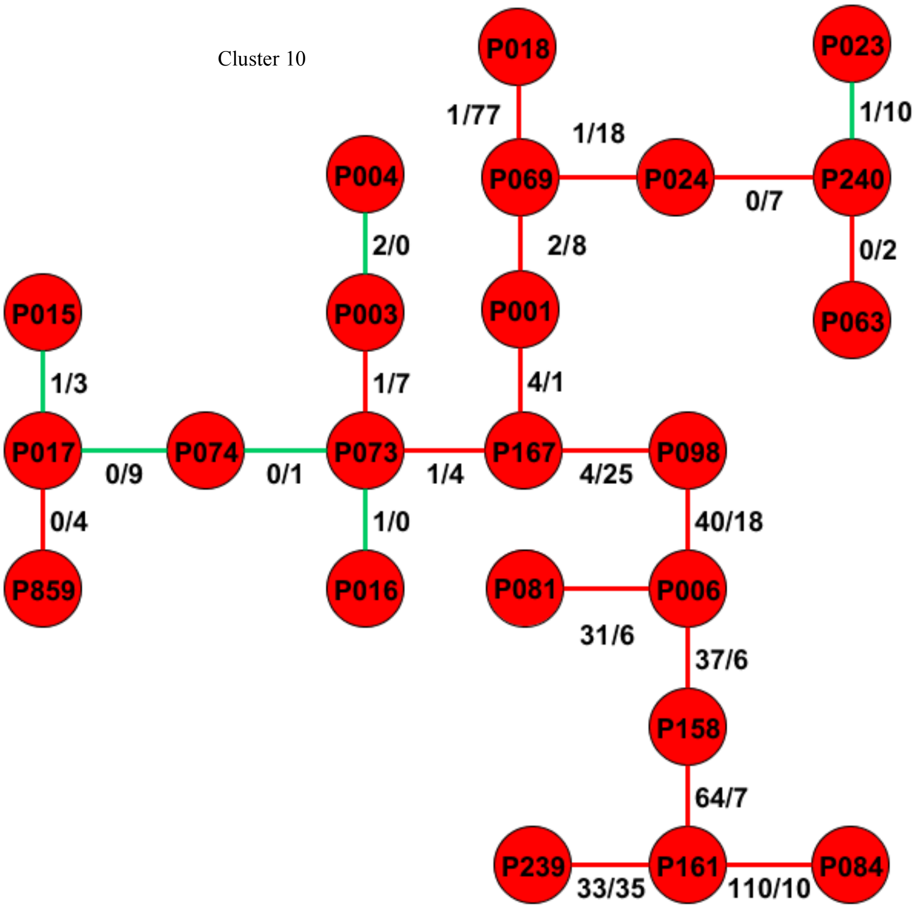

Cluster 11

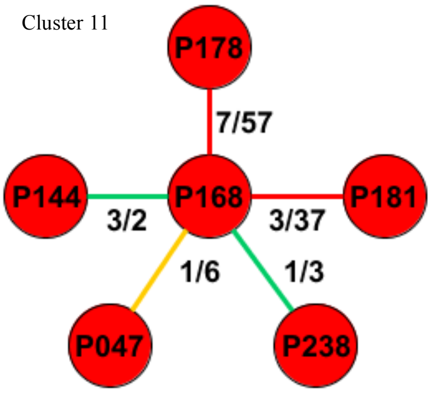

Supplement: Supplementary appendix [file mmc1.pdf]
